# Extinction of all infectious HIV in cell culture by the CRISPR-Cas12a system with only a single crRNA

**DOI:** 10.1093/nar/gkaa226

**Published:** 2020-04-13

**Authors:** Zongliang Gao, Minghui Fan, Atze T Das, Elena Herrera-Carrillo, Ben Berkhout

**Affiliations:** 1 Laboratory of Experimental Virology, Department of Medical Microbiology, Amsterdam UMC, Academic Medical Center, University of Amsterdam, Amsterdam, the Netherlands; 2 Department of Life and Environmental Sciences, University of Cagliari, Italy

## Abstract

The CRISPR-Cas9 system has been used for genome editing of various organisms. We reported inhibition of the human immunodeficiency virus (HIV) in cell culture infections with a single guide RNA (gRNA) and subsequent viral escape, but complete inactivation of infectious HIV with certain combinations of two gRNAs. The new RNA-guided endonuclease system CRISPR-Cas12a (formerly Cpf1) may provide a more promising tool for genome engineering with increased activity and specificity. We compared Cas12a to the original Cas9 system for inactivation of the integrated HIV DNA genome. Superior antiviral activity is reported for Cas12a, which can achieve full HIV inactivation with only a single gRNA (called crRNA). We propose that the different architecture of Cas9 versus Cas12a endonuclease explains this effect. We also disclose that DNA cleavage by the Cas12a endonuclease and subsequent DNA repair causes mutations with a sequence profile that is distinct from that of Cas9. Both CRISPR systems can induce the typical small deletions around the site of DNA cleavage and subsequent repair, but Cas12a does not induce the pure DNA insertions that are routinely observed for Cas9. Although these typical signatures are apparent in many literature studies, this is the first report that documents these striking differences.

## INTRODUCTION

The ability to add, remove, or change DNA sequences is essential to studies that investigate how genetics cause certain phenotypic traits. With its unprecedented efficiency and ease of use, DNA editing technology based on the prokaryotic CRISPR (clustered regularly interspersed short palindromic repeats) Cas9 system is revolutionizing genome engineering (1,2). We and others used CRISPR strategies to target the DNA genome of the human immunodeficiency virus (HIV) (3–9). This pathogenic virus causes a persistent infection that can be controlled by antiretroviral drugs, but a cure is never reached. HIV can persist because it deposits a DNA copy of its genome into that of the host cell, the so-called integrated HIV provirus that frustrates cure attempts. Several laboratories reported HIV inhibition in diverse experimental settings (3–8). However, we demonstrated viral escape when the original *Streptococcus pyogenes* Cas9 (SpCas9) system was instructed to cleave the integrated HIV genome by a single guide RNA (gRNA) (6). CRISPR-mediated cleavage of the target gene leads to its inactivation by the introduction of small indels (insertions or deletions) during DNA repair. Interestingly, the non-homologous end joining (NHEJ) DNA repair mechanism of the host cell is also responsible for the mutations in the viral genome that facilitated HIV escape. Combinations of two gRNAs were subsequently tested and we identified two unique gRNA combinations that trigger full virus inactivation in an infected T cell line: the cure in a bottle (10).

All anti-HIV studies thus far are based on the original Cas9 system. CRISPR system improvements were recently described that improve the efficiency, specificity and therapeutic potential (11–13). We decided to test HIV cure strategies with the novel Cas12a system (LbCas12a from *Lachnospiraceae bacterium*) that seems to exhibit several advantages over the original Cas9-gRNA system (14,15). For instance, Cas12a was reported to have increased target sequence specificity and reduced off-targeting potential (15,16). Furthermore, the gene encoding the Cas12a endonuclease (∼3.7-kb for LbCas12a) and the matching crRNA (43-nt) are smaller than the components of the Cas9 system (∼4.1-kb for SpCas9 and ∼100-nt for gRNA) (15), which could result in significantly improved titers of the viral vector used for gene transfer (17). These two CRISPR systems have a distinct PAM requirement (TTTN for Cas12a versus NGG for Cas9), which may allow one to choose different targets in the HIV genome and the systems also differ in the actual DNA cleavage event, yielding a blunt end (Cas9) or sticky end with a 5′-overhang (Cas12a). The recent literature contains ample examples of utilization of the Cas12a system, both its use in biological studies and its application towards development of future therapeutics (18–23).

In this study, we measured modest HIV inhibition with Cas12a versus Cas9 in transient transfections, but Cas12a outperformed Cas9 in long-term HIV challenge studies in stably transduced T cells. We argue that differences in the DNA editing event directed by the Cas9 versus Cas12a endonuclease explain these differences. In the course of these anti-HIV studies, we also revealed a striking difference in the mutational profile caused by Cas9 versus Cas12a editing. Whereas small indels are known to dominate at Cas9-edited sites, pure sequence insertions were strikingly absent from Cas12a-edited sites. Instead, a new mutation class (termed delin) was revealed that is unique for Cas12a-edited sites. The relevance of this finding and the implications for basic research and therapeutic applications will be discussed.

## MATERIALS AND METHODS

### Plasmid construction

The lentiviral plasmid pY109 (LentiCpf1, addgene# 84740) that harbors the Cas12a gene and crRNA expression cassette was obtained from Feng Zhang (24). A 3′-terminal hepatitis delta virus (HDV) ribozyme, which facilitates precise crRNA processing and results in improved Cas12a activity, was included in the crRNA expression cassette (25). Specifically, a gBlock gene fragment (IDT, Coralville, IA, USA) encoding U6 promoter-control crRNA-HDV was inserted into the XhoI and PacI restriction enzyme sites of pY109 to create plasmid pY109-HDV that contains two BsmBI sites for crRNA cloning. The control crRNA (GGAGACGATATATCGTCTCGCAC) does not target HIV DNA or the human genome. Oligonucleotides encoding HIV targeting crRNAs were ligated into the BsmBI sites of pY109-HDV vector. All crRNAs were designed with the online Benchling software (https://www.benchling.com/crispr/) and are listed in Supplementary Table S1. The crRNA targets with top specificity score were prioritized and the variability of each crRNA target sequence among HIV isolates was estimated by Shannon entropy (Supplementary Table S1). The plasmid pLAI encodes the HIV primary virus isolate LAI (subtype B).

### Cell culture, transfection and transduction

Human embryonic kidney (HEK) 293T cells were cultured in DMEM (Life Technologies, Invitrogen, Carlsbad, CA, USA) supplemented with 10% fetal calf serum (FCS), penicillin (100 U/ml) and streptomycin (100 mg/mL) in a humidified chamber at 37°C and 5% CO_2_. SupT1 T cells (ATCC CRL-1942) were grown in advanced RPMI (GIBCO BRL, Carlsbad, CA, USA) supplemented with l-glutamine, 1% FCS, penicillin (30 U/ml) and streptomycin (30 mg/ml). For transient Cas12a/crRNA activity assays, HEK293T cells (at ∼80% confluence in a 12-well plate) were transfected with 700 ng of pY109-HDV plasmid and 350 ng of pLAI using Lipofectamine 2000 according to the manufacturer's instructions. Two days post-transfection, culture supernatant was collected for CA-p24 ELISA to measure virus production.

Production of lentiviral vectors in HEK293T cells and subsequent transduction of SupT1 T cells was conducted as previously described (17). Briefly, HEK293T cells (at ∼80% confluence in a six-well plate) were transfected with the lentiviral plasmid pY109-HDV and packaging plasmids pSYNGP, pRSV-rev and pVSV-g using Lipofectamine 2000. Two days post-transfection, the lentiviral vector containing supernatant from three wells was centrifuged at low speed, filtered (0.45 μm) and concentrated to 200 μl using the Lenti-X Concentrator Kit according to the manufacturer's protocol (TaKaRa). SupT1 cells (4 × 10^5^ cells in 1 ml of culture medium) were transduced with the concentrated lentiviral vectors. After transduction, the cells were cultured in the presence of puromycin (1 μg/ml) for 10 days to select SupT1 cells expressing Cas12a and an individual crRNA.

### HIV infection and proviral sequence analysis

CRISPR-transduced SupT1 cells (2 × 10^5^ cells in 1 ml of culture medium) were infected with an equal amount of HIV LAI virus corresponding to 1 ng of CA-p24. Cells were passaged twice a week and kept up to 60 days post-infection. Virus spread was monitored by scoring the formation of syncytia every 3 or 4 days. All crRNAs were initially able to suppress virus replication compared to the control cultures, but for some viruses escape was apparent at a later time point (‘breakthrough replication’), whereas other crRNAs were apparently able to continuously suppress HIV replication up to the end of this experiment (‘candidate cured cultures’).

To analyze the candidate escape viruses, the culture supernatant was passaged onto fresh crRNA-transduced SupT1 cells to confirm the escape phenotype. Total cellular DNA (with integrated HIV proviruses) was isolated at the peak of the secondary infection with the QIAGEN DNAeasy kit and worked up for sequencing (see below). For cured cultures that did not demonstrate breakthrough virus replication, we first confirmed the absence of any replication-competent virus by mixing a culture sample with an equal number of control (non-transduced) SupT1 cells, followed by culturing for 30 days to monitor the formation of virus-induced syncytia.

To analyze the integrated HIV proviruses of the candidate cured cultures for the presence of inactivating mutations, we sequenced the crRNA-targets. The infected SupT1 cell cultures were collected at 30 and 60 days post-infection and total cellular DNA was isolated with the QIAGEN DNAeasy kit. The crRNA target regions were amplified by PCR (primers listed in Supplementary Table S2). The PCR products were gel-purified, cloned in the TA-cloning vector and multiple TA-cloned fragments were analyzed by Sanger sequencing. The sequencing reads were aligned with the wild-type (WT) HIV DNA sequence in pLAI.

### Indel pattern analysis for Cas12a and Cas9

In the current Cas12a study, transduced SupT1 cell cultures were challenged with HIV and kept up to 60 days post-infection. Day 30 and 60 samples of the cultures that did not show ‘breakthrough virus replication’ were analyzed for mutations at the Cas12a targets in the integrated HIV provirus. The provirus analysis of the previous Cas9 study (in SupT1 cells collected at day 12 and 110 post-infection), listed in Supplementary Figure S2, was reported in our previous publication (10).

### Literature survey of Cas12a-edited DNA sequences

A PubMed search for manuscripts with the key words ‘Cpf1 and Cas12a’ was performed in early March 2020. We found some 55 papers that provided the actual sequence of Cas12a-edited DNA sites, which are listed in Supplementary Table S3.

## RESULTS

### Targeting the HIV DNA genome by Cas12a/crRNA

**Figure 1.**
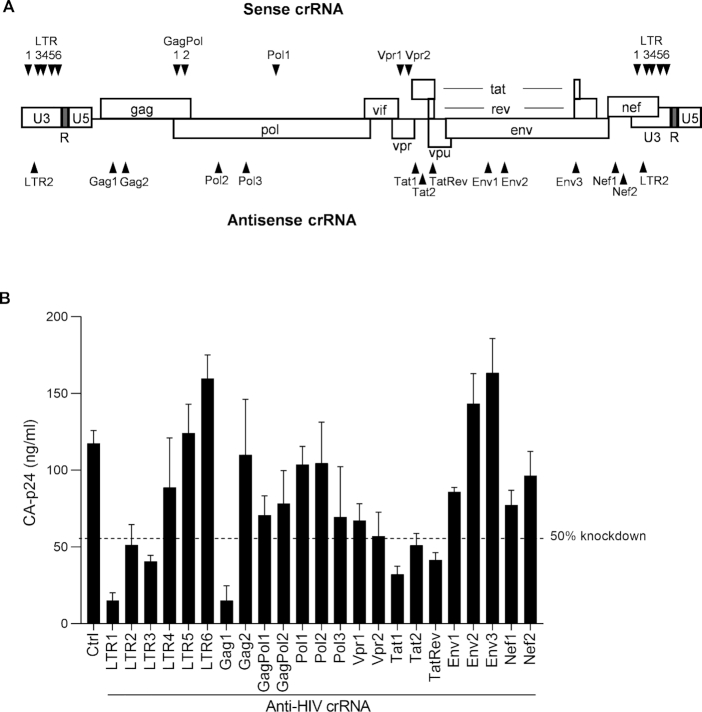
Targeting the HIV genome with single crRNAs. (**A**) The crRNAs were designed to target the sense or antisense strand of HIV proviral DNA. (**B**) The activity of anti-HIV crRNAs to silence HIV DNA. Plasmids encoding HIV LAI and Cas12a/crRNA were co-transfected into HEK293T cells. Two days post-transfection, culture supernatant was collected for CA-p24 ELISA to measure viral gene expression. The dash line reflects 50% knockdown based on the CA-p24 value generated from the Ctrl crRNA that does not target HIV. The data represent the mean ± standard deviation (SD) of *n* = 3 independent biological replicates.

We designed multiple crRNA molecules against the sense and antisense strand of the HIV DNA genome of the primary LAI virus isolate (Figure 1A). For the crRNA design we used the Benchling CRISPR Guide Design Software and we selected crRNAs that target relatively conserved HIV sequences. This latter property will not only broaden the therapeutic potential towards other HIV isolates and even distinct HIV subtypes (26), but will also restrict the likelihood of viral escape as less sequence variation is usually allowed in conserved HIV domains (27). In total, we designed 10 sense and 13 antisense crRNAs, of which a subset (five sense and one antisense) target both long-terminal repeat (LTR) elements that flank the viral genome. We first performed transient transfections in HEK293T cells with the pLAI molecular clone and plasmids encoding the CRISPR reagents (Cas12a endonuclease and crRNA). Virus production was measured by quantitation of the HIV CA-p24 protein in the culture supernatant (Figure 1B). Control (Ctrl) marks unhindered HIV protein expression in the presence of Cas12a and a Ctrl crRNA that targets neither HIV nor the cellular genome. We marked the 50% knockdown threshold to identify the most potent antivirals and we selected 8 crRNAs for further study in T cells to investigate the impact on spreading virus infection: LTR1, LTR2, LTR3, Gag1, Vpr2, Tat1, Tat2 and TatRev.

### HIV inhibition and escape in T cell cultures

**Figure 2.**
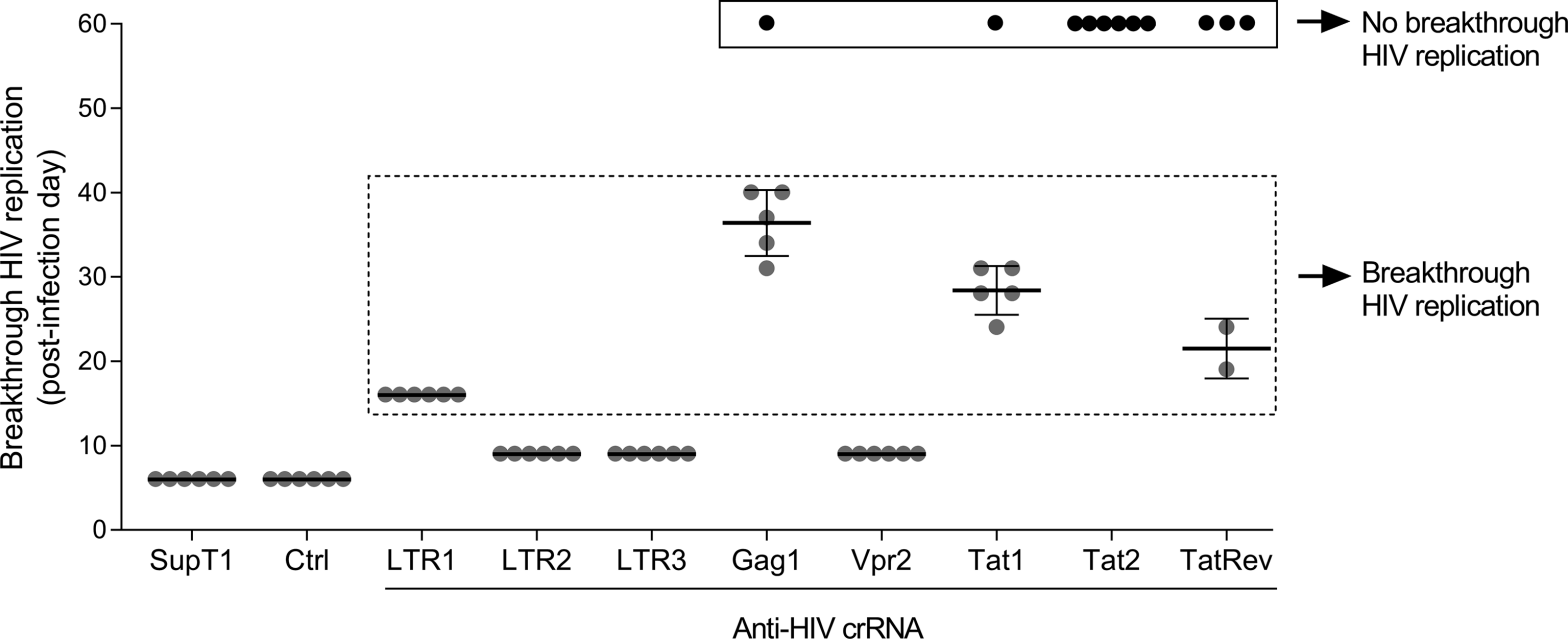
Monitoring HIV replication in Cas12a/crRNA expressing SupT1 cells. Cells were infected with 1 ng CA-p24 HIV (at day 0) and cultured for up to 60 days. The day at which massive virus-induced syncytia was observed (representing breakthrough HIV replication) was scored in 6 parallel cultures after infection (day 0). A single TatRev culture was lost due to bacterial contamination. The cultures without virus replication until day 60 were marked as No breakthrough HIV replication (black box). SupT1, non-transduced cells; Ctrl, cells transduced with a control crRNA that does not target HIV. The data reflects mean values ± SD.

The SupT1 T cell line was stably transduced with lentiviral vectors that encode both CRISPR components (Cas12a endonuclease and a single crRNA) and subsequently infected with the HIV LAI isolate against which the crRNAs were designed. We scored unhindered HIV replication for unprotected SupT1 cells and those transduced with the Ctrl crRNA, yielding massive breakthrough virus replication at day 6 in six independent parallel infections (Figure 2). A very modest antiviral effect was scored for LTR2, LTR3 and Vpr2, indicating that these three crRNAs represent poor inhibitors, leading to massive virus-induced syncytia and high CA-p24 values around day 10 of infection. Potent, but transient HIV inhibition was apparent in most cultures transduced with LTR1, Gag1, Tat1 and TatRev, yielding HIV breakthrough around day 15-40, a pattern that is reminiscent of viral escape (6). Interestingly, several cultures exhibited permanent HIV suppression up to the end of the experiment at day 60 (1× Gag1, 1× Tat1, 3× TatRev, 6× Tat2 cultures). This cure phenotype is most strikingly apparent for all six Tat2-protected cell cultures. Overall, not much overlap was apparent between the results of the transient and stable HIV inhibition studies (Figures 1B and 2, respectively), consistent with previous findings (10). We first analyzed the samples that exhibited potential viral escape and then zoom in on the apparently cured cultures.

**Figure 3.**
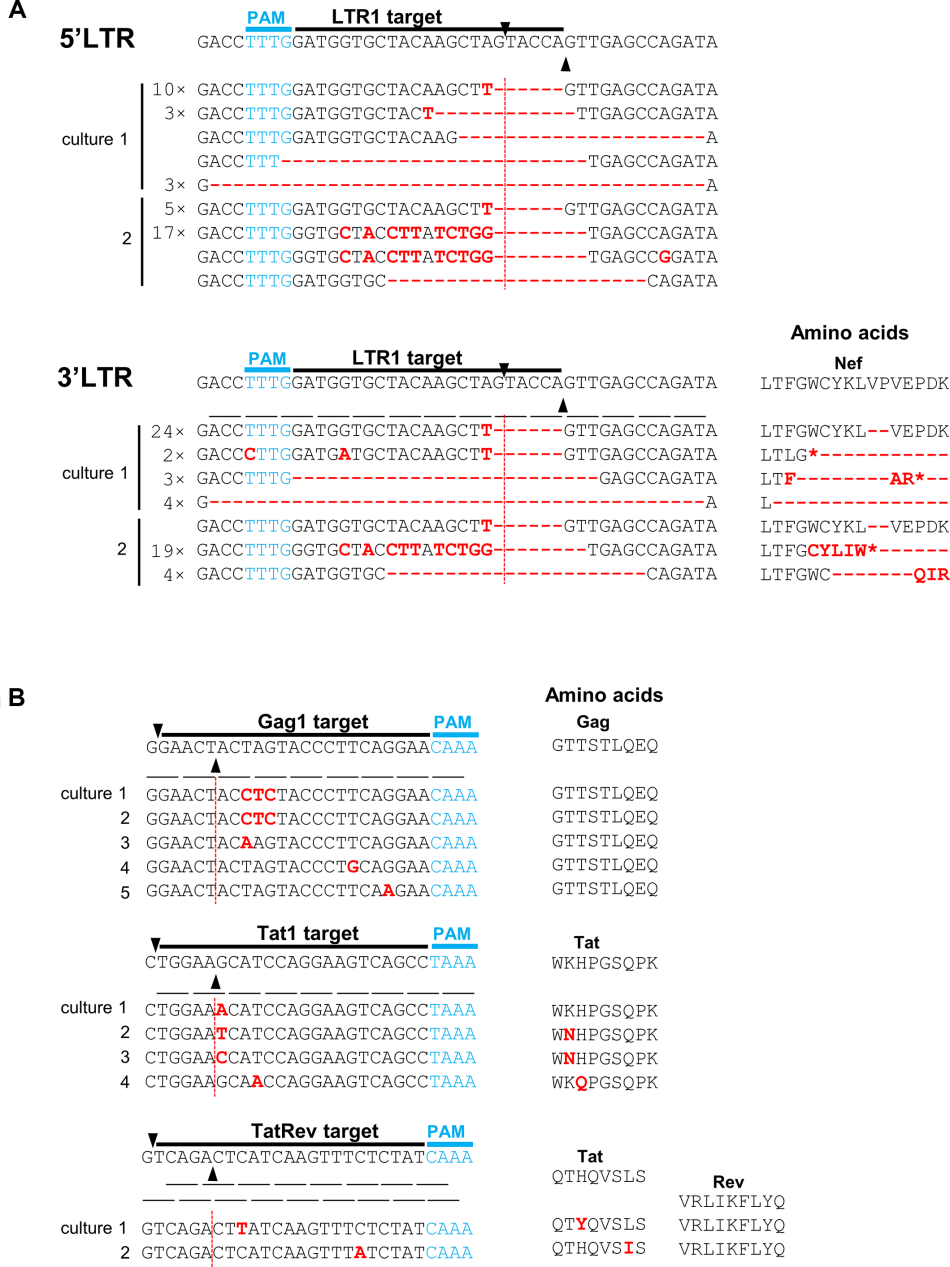
Proviral DNA analysis of breakthrough HIV. (**A**) The LTR1 crRNA instructs Cas12a to target 5′LTR and 3′LTR regions of HIV proviral DNA. Breakthrough virus from two cultures out of six (Figure 2) was subject to a reinfection of LTR1-transduced cells and the cellular DNA was harvested. The LTR1-targeting regions were PCR-amplified and TA-cloned. Multiple TA clones were Sanger-sequenced and subsequently aligned with the input sequence of the HIV LAI isolate, which is the wild-type (WT) sequence shown at the top. The gRNA targets are indicated and the PAM is marked in blue. Black triangles mark the DNA cleavage site. Substitutions, deletions (-) and insertions are highlighted in red. In front of the sequences we indicated those that were detected multiple times. The amino acid codons were indicated below the WT sequence and the amino acid changes (highlighted in red letter or -) were plotted in the left panel. *, stop codon. (**B**) The crRNAs (Gag1, Tat1 and TatRev) guide Cas12a to the viral Gag, Tat and Tat/Rev genes. Breakthrough virus cultures indicated in Figure 2 were analyzed as described in panel A.

One would expect HIV to escape from Cas12a-mediated inhibition by mutation of the target sequence, which can be induced by the error-prone DNA repair process upon DNA cleavage as originally reported for Cas9 (6). The same may indeed be happening for Cas12a as we witnessed the typical small indels around the site of DNA cleavage for LTR1 in both the 5′ and 3′ LTR of the integrated HIV proviruses (Figure 3A). Such indel mutations may not be compatible with HIV replication if they occur in essential parts of the HIV genome, e.g. in critical open reading frames. This likely explains why exclusively less dramatic point-mutations were selected in the targets Gag1, Tat1 and TatRev (Figure 3B). These viral escape events also took longer because not any indel will suffice, meaning that one has to wait longer for the acquisition of relatively unique point-mutations that do not destroy the open reading frame and the activity of the encoded HIV protein. We sometimes observed multiple clustered point-mutations that change multiple codons (Gag1 cultures 1 and 2). These three point-mutations affect two codons, but reflect synonymous codon changes that do not change the encoded amino acids, which may indicate strong evolutionary pressure on the HIV genome to maintain the wild-type protein function (28). Escape from the crRNA-TatRev inhibitor that targets these overlapping genes may cautiously suggest that amino acid substitutions in this genome segment are more easily absorbed by the Tat protein than the Rev protein. The combined results indicate that all observed resistance mutations occur close to the actual site of DNA cleavage, confirming the idea that the error-prone NHEJ DNA repair process is involved in their creation. These results also highlight the exquisite sequence specificity of Cas12a action as single point-mutation seems to cause HIV resistance and viral escape, similar to previous Cas9 results (6,26).

### Candidate cultures in which the HIV infection is cured

We observed durable HIV suppression up to the end of the experiment (day 60) in all six cell cultures equipped with the crRNA Tat2 (Figure 2). The absence of breakthrough HIV replication was also apparent for some Gag1 and Tat1 cultures (each one of the six test cultures) and TatRev (three of five cultures). These events may represent the first signs of a Cas12a-mediated HIV cure *in vitro*. Note that we previously described the same cure result for Cas9, which however took much longer and required the presence of two antiviral gRNAs (10). A direct comparison of the antiviral activity of the two systems is complicated by the fact that they use a different PAM, which makes it impossible to compare the antiviral activity against identical HIV targets.

To document complete HIV inactivation in these cultures, we performed two additional assays. First, we performed an ultra-sensitive virus rescue experiment by addition of WT, unprotected SupT1 cells to a sample of the original cultures that was taken at day 30 and 60 post-infection. Whereas significant virus rescue was apparent for most day 30 samples, all 11 samples taken at day 60 demonstrated a total loss of replication-competent virus (Table 1). Thus, complete HIV inactivation seems to have been achieved in these cases.

**Table 1.** Virus rescue assay

		Virus rescue		WT sequence		Point mutations		Indels	
crRNA	Culture	Day 30	Day 60	Day 30	Day 60	Day 30	Day 60	Day 30	Day 60
Gag1	1	+	−	13	0	3	0	22	38
Tat1	1	−	−	0	2	0	0	30	31
Tat2	1	+	−	10	0	8	0	15	31
	2	+	−	11	0	4	1	11	27
	3	+	−						
	4	+	−						
	5	+	−						
	6	+	−						
TatRev	1	+	−	2	0	22	0	12	32
	2	+	−	20	0	1	0	16	27
	3	+	−						
		Total		56	2	38	1	106	186

Second, we PCR-amplified, TA-cloned and sequenced parts of the integrated HIV proviruses in the original cultures to detect Cas12a-induced mutations that could explain the observed HIV inactivation. An initial Sanger sequencing of the whole PCR product showed that all 11 cultures are mutated around Cas12a targeting sites (data not shown) and we chose 6 representative cultures for TA-cloning to reveal the detailed mutational profile (expanded section of Table 1 and all sequences in Supplementary Figure S1). We simply counted the number of left-over WT proviral sequences versus the number of sequences with a simple (point)mutation or indel over time (Table 1, right 3 columns). Several robust trends are visible. First, we observed a strong loss of WT (56 to 2) and point-mutated (38 to 1) HIV sequences at the expense of viral target sequences with indels (106 to 186). The loss of (point)mutated HIV genomes over time does strongly suggest that these mutants can be recleaved by the Cas12a endonuclease, a phenomenon that was also described for Cas9 (10). Second, it seems that the Tat1 inhibitor is the most rapid HIV inhibitor as the majority of sequences already carry an indel at day 30. Overall, these results provide compelling evidence for complete HIV sterilization by CRISPR-Cas12a by means of only a single antiviral crRNA. Intriguingly, these results also hint at a distinct mutational profile of the Cas12a endonuclease versus the original Cas9 system.

### A distinct mutational profile is induced by Cas12a

**Figure 4.**
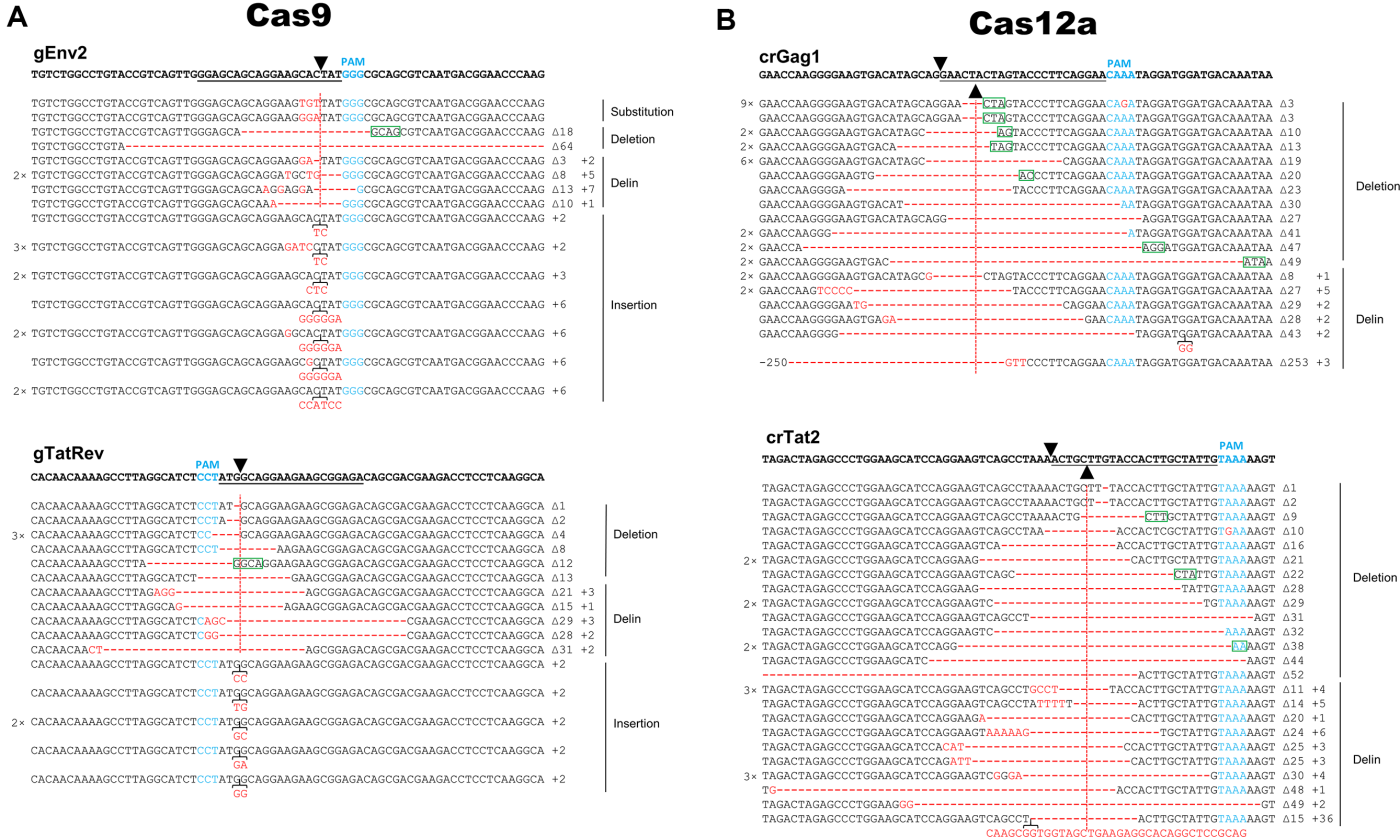
Indel analysis of HIV proviral DNA upon Cas9/Cas12a attack. (**A**) The gRNAs gEnv2 and gTatRev instruct Cas9 to target the Env and Tat/Rev genes, respectively. Cellular DNA was isolated 110 days post HIV infection of Cas9/gRNA-transduced SupT1 T cells. The gRNA-targets in the HIV genome were PCR-amplified, TA-cloned and sequenced. The sequencing reads were aligned with the WT HIV sequence that is shown at the top. The gRNA targets are underlined and the PAM is marked in blue. Black triangles mark the DNA cleavage site. Substitutions, deletions (-) and insertions are shown in red. Sequences were grouped according to their class: substitution, regular deletion or insertion and the new delin class (see the text). In front of the sequences we indicated those that were detected multiple times. Behind each sequence, we listed the size of the actual deletion (Δ) and insertion (+). No unedited WT sequences were detected for these gRNAs. (**B**) The crRNAs crGag1 and crTat2 guide Cas12a to the viral Gag and Tat genes, respectively. Cellular DNA was isolated 60 days post HIV infection of the Cas12a/crRNA-transduced SupT1 T cells and worked up as described in panel A. No unedited WT sequences were detected for these crRNAs. Microhomologies of ≥2 nt at the break sites are marked by a green box.

Close inspection of the genetic lesions induced by Cas12a revealed a striking difference with previous Cas9 results (10). A representative set of sequences is shown in Figure 4, comparing the new Cas12a results with previously reported Cas9 results (10). We plotted HIV sequences around the cleavage site for 2 gRNAs (gEnv2 and gTatRev) of the Cas9 system (Figure 4A) and 2 crRNAs (crGag1 and crTat2) of the Cas12a system (Figure 4B), the latter representing the day 60 data from Supplementary Figure S1. Both CRISPR systems generate the typical small deletions around the cleavage site, but the Cas12a profile differs from the regular indel pattern induced by Cas9 in one notable aspect. Small insertions are readily observed at the cleavage site of the Cas9 products, but this mutation class of ‘pure inserts’ is absent for Cas12a (Figure 4B). We do see small insertions for Cas12a, but exclusively in the context of a deletion. This special mutation class can also be recognized for Cas9. To distinguish these mutational patterns we will use the standard indel terminology for regular (‘pure’) deletions or insertions and we propose the ‘delin’ name for the new mutant class of a small insertion that occurs in the context of a deletion. This typical Cas12a mutational profile lacking regular insertions was also apparent during HIV escape from the LTR1 inhibitor (Figure 3A) and HIV inactivation by other crRNA inhibitors (Supplementary Figure S1). The combined deletion-insertion or delin pattern has - to the best of our knowledge - not been described previously, which is striking given the intense international research ongoing using CRISPR technology. To us, it only became apparent upon close investigation and comparison of the two CRISPR systems.

We wanted to analyze the delin characteristics in more detail, but first will discuss two other events that became apparent from inspection of the sequences presented in Figure 4. First, we sometimes observed identical mutants multiple times. An exceptional example is shown in the top line for crGag1, a 3 bp deletion that was observed 9×. It is possible that sequence amplification occurred during virus replication or work-up of the DNA sample (e.g. during PCR-amplification or TA-cloning). Alternatively, this could represent a mutational hotspot that facilitates DNA end joining, e.g. by short 2-25 bp regions of sequence homology or microhomologies flanking the DNA break (29) (marked as green box in Figure 4). We therefore decided to count each sequence only once in the subsequent analyses. Second, an exceptionally long 36-bp insert is present in one of the crTat2 sequences in combination with a 15-bp deletion (bottom line for crTat2 in Figure 4). Blast analysis of the insert indicated that this sequence represents a perfect copy of a sequence present some 2.3 kb downstream in the Env gene of the HIV genome. This likely represents an atypical recombination event, which is known to occur frequently during HIV replication (30–32). This recombination event has most likely been triggered by CRISPR-mediated DNA cleavage because the insertion, as well as the deletion, occurred precisely at the cleavage site. This unique insert sequence was removed from the subsequent analysis of the general delin characteristics.

**Figure 5.**
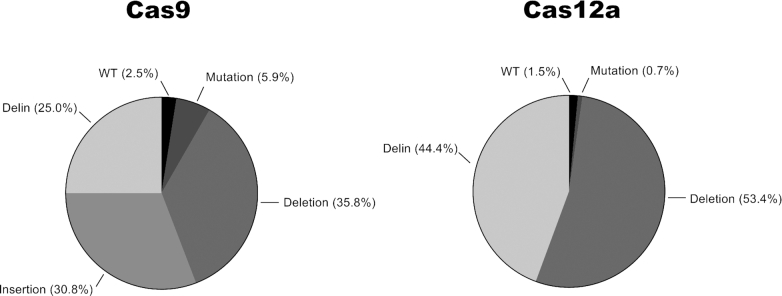
Indel pattern upon CRISPR attack. The frequency of WT HIV target sequences and different types of mutations (regular deletion and insertion, and the new delin class) for Cas9 (left) and Cas12a (right panel). This analysis was performed for HIV-infected T cell cultures that were collected at 110 days (Cas9 attack with two gRNAs) or 60 days (Cas12a attack with an individual crRNA). We analyzed 125 sequences for Cas9 (Supplementary Figure S2) and 114 sequences for Cas12 (Supplementary Figure S1, Figure 3 shows a subset of these sequences).

We calculated the relative frequency of the different mutational events in cultures cured of replicating HIV (Figure 5). This analysis was performed for a set of eight gRNAs (Cas9) and six crRNAs (Cas12a) scattered across the HIV genome, yielding a total of 125 (Cas9) and 114 (Cas12a) analyzed sequences. Similar results were obtained for the different gRNAs, which we will collectively term the Cas9 group. Likewise, the crRNA results could easily be combined as the Cas12a group. For both CRISPR systems we detected a minority of ‘left-over’ WT HIV sequences, which apparently were not yet cleaved and repaired/mutated. More WT sequences are present upon Cas9 attack for 110 days with two gRNAs compared to Cas12a attack with a single crRNA for 60 days, confirming the superior antiviral activity of Cas12a. Importantly, we confirmed the absence of any regular insertions among the Cas12a-mutated sequences. In contrast, 30.8% of the Cas9-generated sequences have a regular insert at the cleavage site. We counted many deletions for Cas9, with regular deletions (35.8%) and delins (25.0%). Both deletion types are also present for Cas12a, but at increased rates (53.4% and 44.4%, respectively), most likely because of the absence of the regular insertion class. For both Cas9 and Cas12a, we noticed a slight preference for regular deletions over delins. This similarity may cautiously suggest that delin formation is intrinsically linked to the DNA repair process and not so much to the initial DNA cleavage event that differs for Cas9 (blunt end) versus Cas12a (sticky end).

**Figure 6.**
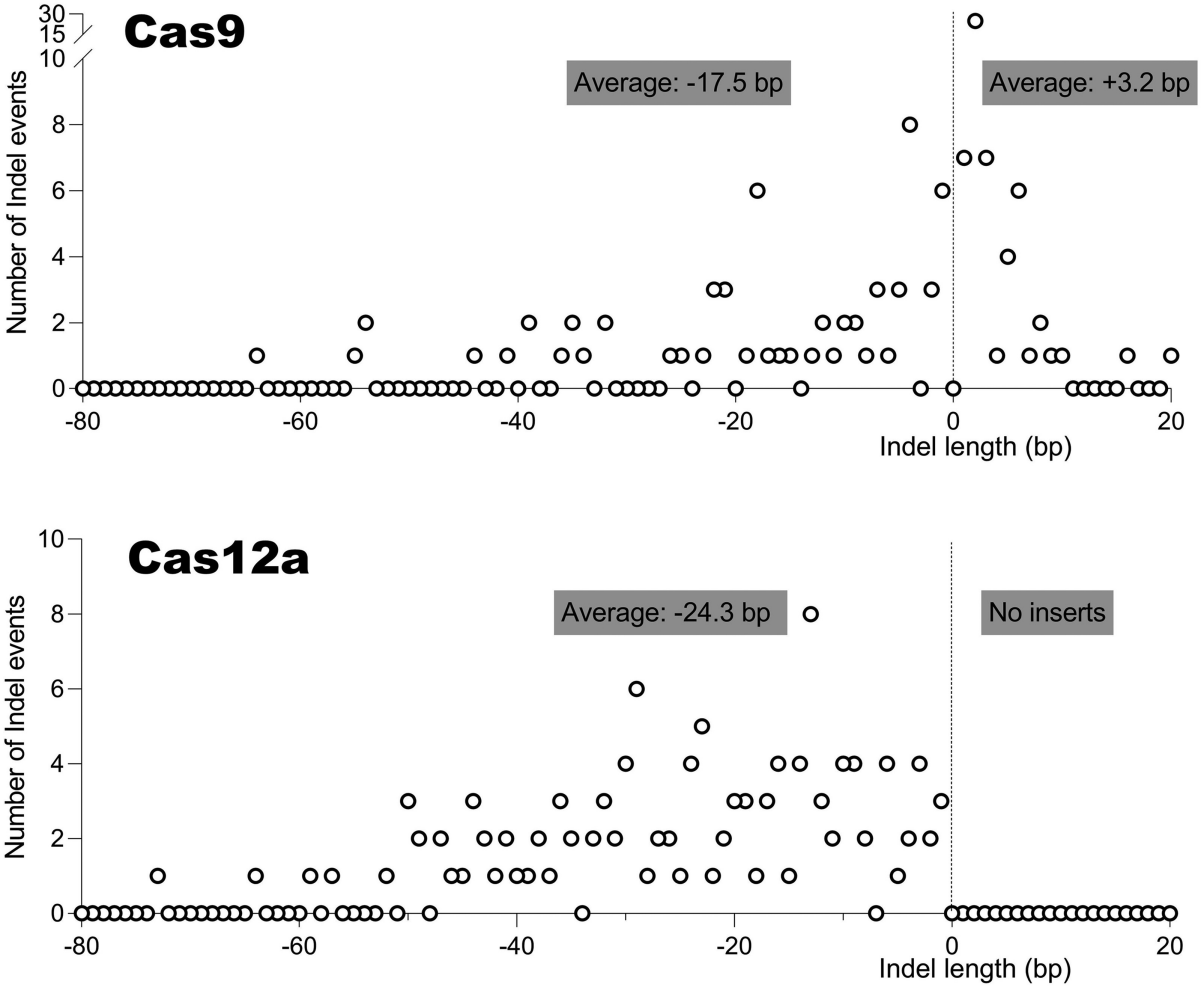
Regular deletions and insertions upon CRISPR attack. Frequency distribution of the size of regular deletions and insertions generated by Cas9 (top) and Cas12a (bottom). A few sequences with much larger deletions (>80 bp) are not shown. The average sizes of the deletions and insertions are indicated.

We next analyzed the size of the regular deletions and insertions in more detail (Figure 6). For this analysis, we collected all sequences obtained during these Cas9 and Cas12a experiments and removed duplicate sequences for the reason presented above, which resulted in a total of 190 (Cas9) and 243 (Cas12a) sequences. This survey confirms the absolute absence of regular insertions for Cas12a. We calculated the average deletion and insertion lengths (indicated in graphs). The Cas9 products show an average deletion of 17.5 bp and an average insertion of 3.2 bp. The Cas12 products exhibit a larger deletion size of 24.3 bp and - as said - no regular insertions.

**Figure 7.**
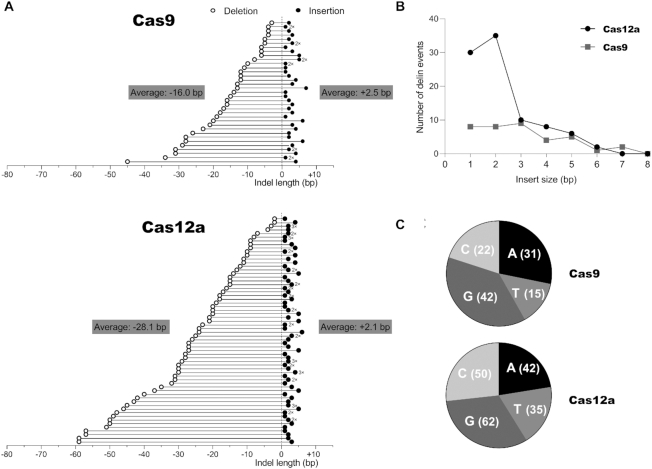
Analysis of the new delin mutation class. (**A**) Size of the deletion and insertion component in delins. We analyzed the delin class generated by Cas9 (top) and Cas12a (bottom). The size (in bp) of the combined deletion (open circle) and insertion (closed circle) is plotted to the left/right of the Y-axis. For Cas12a, we observed two larger deletions (> 80 bp) that are not plotted. The average sizes were calculated for all plotted data points. Distinct delins with the same sizes of the deletion-insertion are marked (e.g. 2×). (**B**) Size of delin inserts. Frequency distribution of the insert size (in bp) of the delin class of mutants. (**C**) Nucleotide composition of delin inserts. This analysis was performed for the positive or sense strand of the HIV target sequence.

We also analyzed the delin class of mutants in more detail for both CRISPR systems (Figure 7). In a few cases, different delins were observed with - by chance - identical sizes of the deletion and insertion components. These cases are marked by 2× and 3× signs. For both CRISPR systems, it is immediately clear that the deletion-component varies considerably in size, whereas the insertion-component is usually much shorter. Consequently, there is no correlation between the sizes of the deletion and insertion components of the delins. The average size of the deletion and insertion component of the delins was calculated and is shown as inserts in Figure 7A. The deletion component in Cas12a-induced delins is significantly larger than that of Cas9 delins (average size 28.1 and 16.0 bp, respectively), a trend that is similar to the regular deletions shown in Figure 6 (-24.3 bp versus -17.5 bp). Cas9-induced insertions are similarly small in the delin class (+2.5 bp) versus the regular insertions (+3.2 bp). Such a comparison cannot be performed for Cas12a as this system does not generate any regular insertions.

We also plotted the distribution of the insert size (Figure 7B). The maximum size of the insert is similar for Cas9 (6 bp) and Cas12a (7 bp), but Cas12a seems to prefer very small inserts of 1 and especially 2 bp. The small delin inserts generally contain all four possible nucleotides as plotted for the sense strand of the DNA target (Figure 7C). Such detailed characteristics were not analyzed in the original Cas12a study (15), but inspection of the published sequences confirms the absence of regular insertions among the 10 provided sequences, which is fully consistent with our current findings.

## DISCUSSION

CRISPR-Cas9 demonstrated its ability to inhibit HIV replication both in cultured cells and in an animal model, but complete HIV inactivation (functional cure) has never been realized with a single gRNA (3–10). Viral escape usually occurs when a single antiviral gRNA is used. Certain combinations of two gRNAs could achieve complete HIV inactivation (10), but this strategy also has some downsides. For instance, the simultaneous use of two gRNAs increases off-targeting and genome rearrangement potential (33). Introducing additional gRNAs also makes CRISPR delivery more difficult, especially when viral vectors with a limited packaging capacity are used. For example, the inclusion of more gRNA expression cassette hampers the packaging in Adeno-associated virus (AAV) vectors and reduces the titer of lentiviral vectors (33,34). Thus, the use of a single gRNA would be therapeutically advantageous in a HIV gene therapy setting. In this study, we report complete HIV sterilization by CRISPR-Cas12a with a single crRNA *in vitro* in a T cell line, providing the most powerful CRISPR tool for HIV inactivation reported so far.

**Figure 8.**
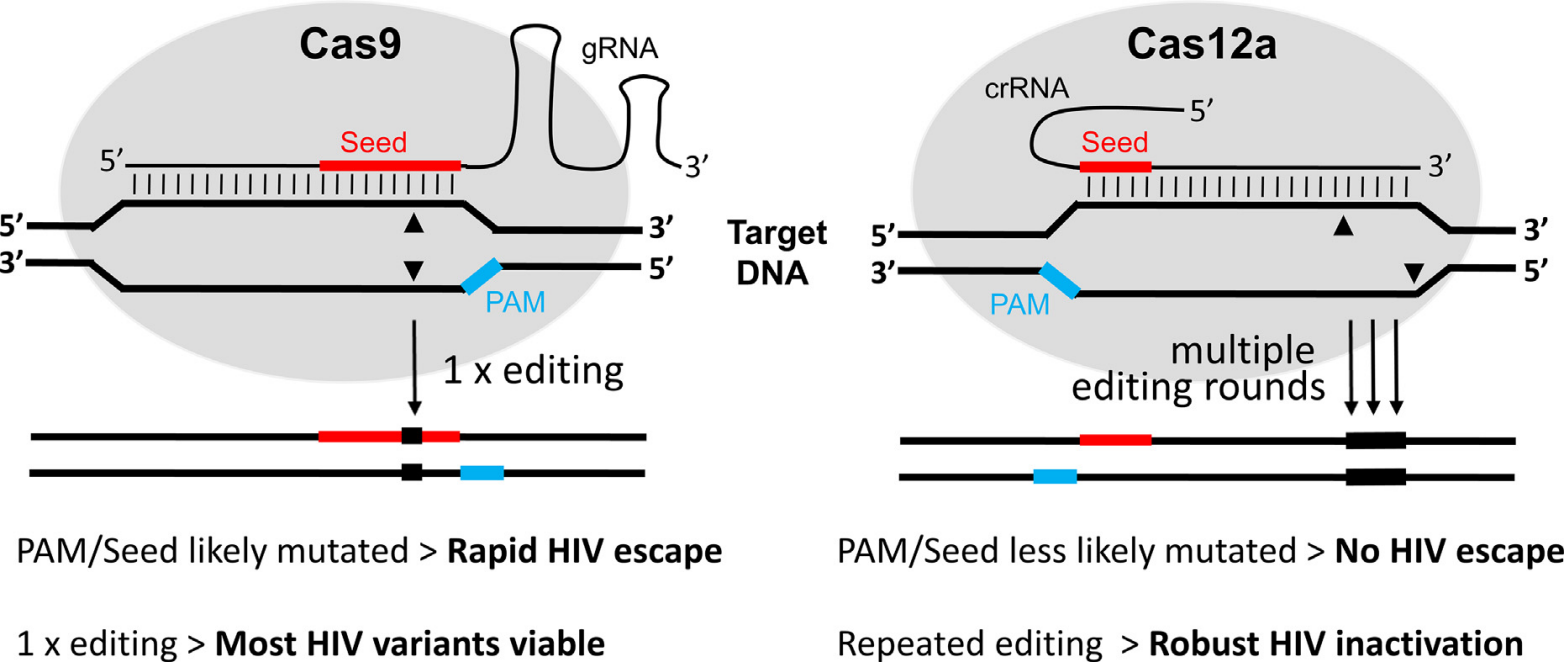
Schematic of target DNA recognition and cleavage by the Cas9 versus Cas12a endonuclease. The Seed region (red) of the guide is a ∼10-nt and 5-nt sequence close to the PAM for the gRNA and crRNA, respectively (15,44). The PAM is marked in blue and the cleavage sites are indicated by triangles. In brief, Cas9 editing will likely change the proximal PAM/Seed sequence, thus triggering HIV escape and blocking repeated editing events that may lead to complete inactivation of the HIV genome. Cas12a is less likely to alter the more distal PAM/Seed, thus avoiding HIV escape. But repeated editing is now possible on the unaltered PAM/Seed, thus leading to HIV inactivation. See the text for further details.

Despite these superior HIV cure results for Cas12a compared to Cas9, Cas12a demonstrated only modest HIV inhibition in transient transfection assays. The relative ability of Cas12a to restrict viral escape may be linked to the difference in architecture of the Cas12a versus Cas9 endonuclease (Figure 8). For Cas9, mutations introduced upon DNA cleavage/repair will likely affect the important and overlapping ‘Seed sequence’ and the proximal PAM motif. This mutational event will trigger HIV escape, also because repeated editing is less likely as the PAM/Seed will be affected. For Cas12a, these mutations will end up in DNA sequences that are less critical for editing, thus allowing repeated editing cycles of DNA cleavage and subsequent repair. No HIV escape is triggered because the PAM/Seed sequences that are important for editing remain intact. But the repeated rounds of editing will affect HIV viability because our CRISPR reagents target well-conserved viral sequences that are important for HIV replication fitness (28). This explains the sustained antiviral activity of Cas12a over Cas9 in stably transduced T cell lines, whereas the latter seemed more potent in transient transfection assays. Despite the superior cure activity of Cas12a, one could consider the development of a double crRNA attack as this may trigger more complete HIV inactivation, e.g. by excision of the intervening HIV sequences (35).

In the course of this study, we recognized a differential mutational profile of Cas12a versus Cas9. Both systems induce regular deletions and deletions with small insertions at the end (which we termed delins), but Cas12a lacks the ability to generate pure insertions. In fact, this typical mutation profile is apparent in the original Cas12a study, but was not recognized (15). As Cas12a has become a popular editing tool, we screened the literature for the type of Cas12a-introduced lesions. This survey identified some 55 papers that in total reported some 1569 altered target sequences. We grouped the studies based on the organism under study (mammalian, plants etc.) and counted the number of standard deletions and insertions, but also the new delin class (Supplementary Table S3). This survey strongly endorsed our Cas12a findings: a notable absence of regular insertions and a prevalence of delins. We also tabulated the size of these delins, which globally aligns with our current observations. Some 124 delins were present among the 1569 sequences (average 7.9%), but with large differences between studies. Some of the larger studies (references 17 and 21 in Supplementary Table S3) reported a roughly equal occurrence of deletions versus delins, similar to our findings. We realize that one should be careful with such calculations as some studies may have filtered out unexpected sequences, e.g. the ones with the characteristic delin pattern.

**Figure 9.**
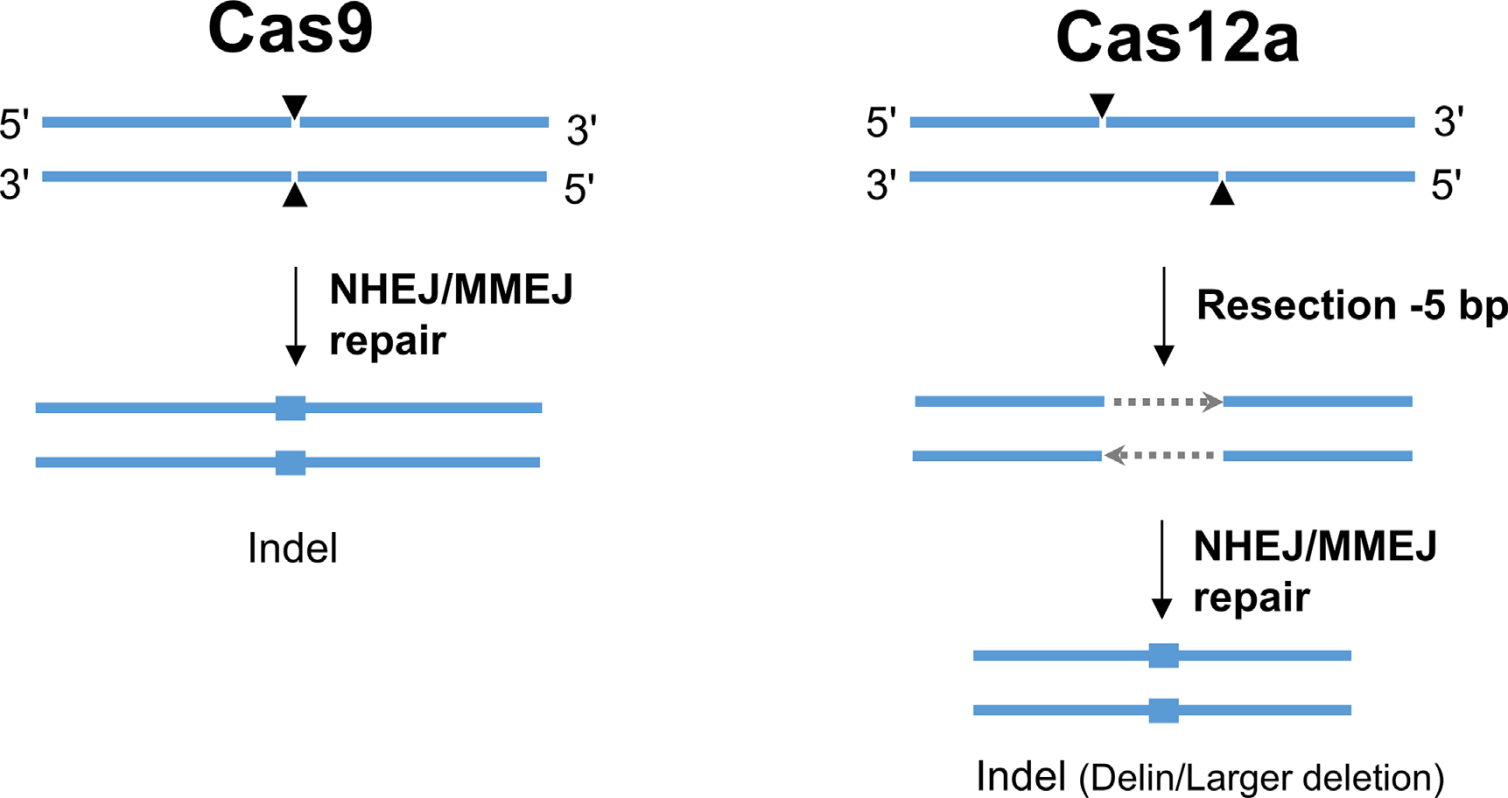
Model for differential DNA repair routes induced by Cas9 versus Cas12a DNA cleavage. See the discussion for further explanation.

This difference in mutational profile of Cas12a versus Cas9 is likely caused by the different DNA lesion that is introduced by these endonucleases. Whereas Cas9 generates blunt DNA ends, Cas12a yields a staggered cut with a 5′-overhang of 5 nucleotides. In the absence of a homologous recombination donor template, Cas endonuclease-induced DNA double-strand breaks (DSBs) are repaired by two major pathways: NHEJ and microhomology-mediated end joining (MMEJ). The latter is also known as the alternative NHEJ (Alt-NHEJ) pathway that relies on microhomology sequences of 2-25 bp (29,36–38). We actually observed microhomology sequences as hallmarks of MMEJ repair in both the Cas9- and Cas12a-edited sites (green boxes in Figure 4). This initial survey suggests that Cas12a induces more MMEJ events (70/175) than Cas9 (16/78). The DNA repair process is complex and requires multiple enzymes for processing of the DNA ends by nucleases, gap fill-in activity of DNA polymerases and a final ligation step (39). Processing of DSB ends via 5′-3′ resection, which requires nucleolytic degradation of the 5′-terminated strands to generate 3′-single stranded DNA (ssDNA) tails, seems to be a major step during DNA repair by NHEJ and MMEJ. Here we propose a model for the distinct repair outcomes at Cas9- and Cas12a-induced DSB based on what is known for DNA repair pathways (Figure 9). NHEJ repair does not require resection, but may need nucleases such as Artemis for DNA trimming to generate compatible blunt ends for ligation. Intriguingly, Artemis has a preferential resection activity for DNA ends with 5′ overhangs (40), which matches the product generated by Cas12a. It is thus possible that Artemis is more frequently involved in Cas12a-based genome editing than Cas9-editing. Different from NHEJ, MMEJ utilizes a 5′-3′ end-resection to reveal short homologies on each stands of the DSB for initiation of DNA repair. This resection involves a 5′ to 3′ exonuclease such as Mre11 and CtIP to generate 3′-ssDNA. Therefore, in MMEJ repair, one could imagine that the 5-nt 5′ overhangs of the Cas12a product is resected before the 3′ overhang is exposed, rendering a 5-nt bigger resection than the blunt ended Cas9 product (Figure 9). Moreover, a recent study showed that DSBs with 5′ overhangs undergo more dramatic resection, which skews DNA repair towards the Alt-NHEJ pathway, indicating that DSBs with 5′ overhangs are better substrate for resection (41). Thus, different DNA-end configurations trigger the assembly of different DNA repair enzymes that leave distinct mutations at the cleavage and repair site (39–41). DNA ends with overhangs are subject to more extensive DNA end resection by exonuclease or endonuclease activity than blunt DNA ends (40,41). As a consequence, the repair of staggered DNA lesions will likely endure a greater sequence loss than blunt end lesions. Indeed, repair of Cas12a-induced staggered cuts introduced deletions more frequently (Figure 5) and indels with a larger average size (Figure 6) than observed for the blunt ends induced by Cas9. The insertion component of the delin mutations is very similar for Cas12a and Cas9, both in size and nucleotide-composition (Figure 7), indicating that the insertion process is not sensitive to the type of DNA lesion. In contrast, regular insertions are unique for Cas9 and absent for Cas12a. This may be due to the fact that the Cas12a product is resected, resulting in a 5-nt loss prior to regular nucleotide insertion/deletion during DNA repair (Figure 9). This notable difference in mutational profile may explain the superior HIV cure results obtained for Cas12a.

The current Cas12a findings may obviously bear relevance for other CRISPR gene editing studies. There could be research questions or applications where nucleotide insertions are not desired. The Cas12a system may be ideal for such projects as no regular insertions are introduced. For example, Cas12a may be helpful in research strategies that aim to avoid the creation of neo-epitopes in protein-encoding genes. On the other hand, Cas12a (and Cas9) will produce the delin mutant class, consisting of a small insertion within a deletion. As the average insertion in Cas12a-delins is only 2.1 bp, this will add at most a single codon and thus a single additional amino acid. Cas12a may also outperform Cas9 in strategies designed to disrupt the function of a gene as a somewhat larger deletion is induced. We indeed observed more robust HIV inhibition with Cas12a than Cas9, but think that this is primarily due to repeated Cas12a cleavage as the Seed sequence likely remains intact (Figure 8). Together with the merits listed in the introduction, Cas12a provides an attractive genome editing platform with distinct properties.

An early Cas9 study indicated that the indel pattern is non-random with individual target sites showing a preference for the type of mutation (insertion or deletion) and indel size (42). A recent Cas9 study revealed that the mutation pattern upon repair of the cleaved DNA can in fact be predicted based on the actual sequence of the target site (43). This finding has important practical implications as it may allow one to steer the editing process in the desired direction (e.g. gene inactivation or sequence insertion). It may thus be important to test whether such predictions can also be unveiled for the Cas12a system.

## Supplementary Material

gkaa226_Supplemental_Files

## Notes

Present address: Zongliang Gao, Department of Biomedicine, Aarhus University, Aarhus, Denmark.

## References

[1] Doudna J.A., Charpentier E. Genome editing. The new frontier of genome engineering with CRISPR-Cas9. Science. 2014; 346:1258096. 25430774 10.1126/science.1258096

[2] Hsu P.D., Lander E.S., Zhang F. Development and applications of CRISPR-Cas9 for genome engineering. Cell. 2014; 157:1262–1278. 24906146 10.1016/j.cell.2014.05.010 PMC4343198

[3] Ebina H., Misawa N., Kanemura Y., Koyanagi Y. Harnessing the CRISPR/Cas9 system to disrupt latent HIV-1 provirus. Sci. Rep. 2013; 3:2510. 23974631 10.1038/srep02510 PMC3752613

[4] Hu W., Kaminski R., Yang F., Zhang Y., Cosentino L., Li F., Luo B., Alvarez-Carbonell D., Garcia-Mesa Y., Karn J. et al. RNA-directed gene editing specifically eradicates latent and prevents new HIV-1 infection. Proc. Natl Acad. Sci. U.S.A. 2014; 111:11461–11466. 25049410 10.1073/pnas.1405186111 PMC4128125

[5] Liao H.K., Gu Y., Diaz A., Marlett J., Takahashi Y., Li M., Suzuki K., Xu R., Hishida T., Chang C.J. et al. Use of the CRISPR/Cas9 system as an intracellular defense against HIV-1 infection in human cells. Nat. Commun. 2015; 6:6413. 25752527 10.1038/ncomms7413

[6] Wang G., Zhao N., Berkhout B., Das A.T. CRISPR-Cas9 can inhibit HIV-1 replication but NHEJ repair facilitates virus escape. Mol. Ther. 2016; 24:522–526. 26796669 10.1038/mt.2016.24 PMC4786927

[7] Wang Z., Pan Q.H., Gendron P., Zhu W.J., Guo F., Cen S., Wainberg M.A., Liang C. CRISPR/Cas9-derived mutations both inhibit HIV-1 replication and accelerate viral escape. Cell Rep. 2016; 15:481–489. 27068471 10.1016/j.celrep.2016.03.042

[8] Yin C., Zhang T., Qu X., Zhang Y., Putatunda R., Xiao X., Li F., Xiao W., Zhao H., Dai S. et al. In vivo excision of HIV-1 provirus by saCas9 and multiplex single-guide RNAs in animal models. Mol. Ther. 2017; 25:1168–1186. 28366764 10.1016/j.ymthe.2017.03.012 PMC5417847

[9] Dash P.K., Kaminski R., Bella R., Su H., Mathews S., Ahooyi T.M., Chen C., Mancuso P., Sariyer R., Ferrante P. et al. Sequential LASER ART and CRISPR treatments eliminate HIV-1 in a subset of infected humanized mice. Nat. Commun. 2019; 10:2753. 31266936 10.1038/s41467-019-10366-y PMC6606613

[10] Wang G., Zhao N., Berkhout B., Das A.T. A combinatorial CRISPR-Cas9 attack on HIV-1 DNA extinguishes all infectious provirus in infected T cell cultures. Cell Rep. 2016; 17:2819–2826. 27974196 10.1016/j.celrep.2016.11.057

[11] Tycko J., Myer V.E., Hsu P.D. Methods for optimizing CRISPR-Cas9 genome editing apecificity. Mol. Cell. 2016; 63:355–370. 27494557 10.1016/j.molcel.2016.07.004 PMC4976696

[12] Komor A.C., Badran A.H., Liu D.R. CRISPR-based technologies for the manipulation of eukaryotic genomes. Cell. 2017; 169:559. 10.1016/j.cell.2017.04.005 28431253

[13] Bak R.O., Gomez-Ospina N., Porteus M.H. Gene editing on center stage. Trends Genet. 2018; 34:600–611. 29908711 10.1016/j.tig.2018.05.004

[14] Fagerlund R.D., Staals R.H., Fineran P.C. The Cpf1 CRISPR-Cas protein expands genome-editing tools. Genome Biol. 2015; 16:251. 26578176 10.1186/s13059-015-0824-9 PMC4647450

[15] Zetsche B., Gootenberg J.S., Abudayyeh O.O., Slaymaker I.M., Makarova K.S., Essletzbichler P., Volz S.E., Joung J., Van Der Oost J., Regev A. et al. Cpf1 is a single RNA-guided endonuclease of a class 2 CRISPR-Cas system. Cell. 2015; 163:759–771. 26422227 10.1016/j.cell.2015.09.038 PMC4638220

[16] Kim D., Kim J., Hur J.K., Been K.W., Yoon S.H., Kim J.S. Genome-wide analysis reveals specificities of Cpf1 endonucleases in human cells. Nat. Biotechnol. 2016; 34:863–868. 27272384 10.1038/nbt.3609

[17] Gao Z.L., Herrera-Carrillo E., Berkhout B. A single H1 promoter can drive both guide RNA and endonuclease expression in the CRISPR-Cas9 system. Mol Ther-Nucl Acids. 2019; 14:32–40. 10.1016/j.omtn.2018.10.016 PMC6288460 30530211

[18] Park H.M., Liu H., Wu J., Chong A., Mackley V., Fellmann C., Rao A., Jiang F., Chu H., Murthy N. et al. Extension of the crRNA enhances Cpf1 gene editing in vitro and in vivo. Nat. Commun. 2018; 9:3313. 30120228 10.1038/s41467-018-05641-3 PMC6098076

[19] Dai X., Park J.J., Du Y., Kim H.R., Wang G., Errami Y., Chen S. One-step generation of modular CAR-T cells with AAV-Cpf1. Nat. Methods. 2019; 16:247–254. 30804551 10.1038/s41592-019-0329-7 PMC6519746

[20] Kleinstiver B.P., Sousa A.A., Walton R.T., Tak Y.E., Hsu J.Y., Clement K., Welch M.M., Horng J.E., Malagon-Lopez J., Scarfo I. et al. Engineered CRISPR-Cas12a variants with increased activities and improved targeting ranges for gene, epigenetic and base editing. Nat. Biotechnol. 2019; 37:276–282. 30742127 10.1038/s41587-018-0011-0 PMC6401248

[21] Li S., Li J., He Y., Xu M., Zhang J., Du W., Zhao Y., Xia L. Precise gene replacement in rice by RNA transcript-templated homologous recombination. Nat. Biotechnol. 2019; 37:445–450. 30886437 10.1038/s41587-019-0065-7

[22] Liu P., Luk K., Shin M., Idrizi F., Kwok S., Roscoe B., Mintzer E., Suresh S., Morrison K., Frazao J.B. et al. Enhanced Cas12a editing in mammalian cells and zebrafish. Nucleic Acids Res. 2019; 47:4169–4180. 30892626 10.1093/nar/gkz184 PMC6486634

[23] Xu S., Luk K., Yao Q., Shen A.H., Zeng J., Wu Y., Luo H.Y., Brendel C., Pinello L., Chui D.H.K. et al. Editing aberrant splice sites efficiently restores β-globin expression in β-thalassemia. Blood. 2019; 133:2255–2262. 30704988 10.1182/blood-2019-01-895094 PMC6533605

[24] Zetsche B., Heidenreich M., Mohanraju P., Fedorova I., Kneppers J., Degennaro E.M., Winblad N., Choudhury S.R., Abudayyeh O.O., Gootenberg J.S. et al. Multiplex gene editing by CRISPR-Cpf1 using a single crRNA array. Nat. Biotechnol. 2017; 35:31–34. 27918548 10.1038/nbt.3737 PMC5225075

[25] Gao Z., Herrera-Carrillo E., Berkhout B. Improvement of the CRISPR-Cpf1 system with ribozyme-processed crRNA. RNA Biol. 2018; 15:1458–1467. 30470168 10.1080/15476286.2018.1551703 PMC6333430

[26] Darcis G., Binda C.S., Klaver B., Herrera-Carrillo E., Berkhout B., Das A.T. The impact of HIV-1 genetic diversity on CRISPR-Cas9 antiviral activity and viral escape. Viruses. 2019; 11:255. 10.3390/v11030255 PMC6466431 30871200

[27] Von Eije K.J., Ter Brake O., Berkhout B. Human immunodeficiency virus type 1 escape is restricted when conserved genome sequences are targeted by RNA interference. J. Virol. 2008; 82:2895–2903. 18077712 10.1128/JVI.02035-07 PMC2258968

[28] Ter Brake O., Von Eije K.J., Berkhout B. Probing the sequence space available for HIV-1 evolution. AIDS. 2008; 22:1875–1877. 18753935 10.1097/QAD.0b013e328309efe3

[29] Sfeir A., Symington L.S. Microhomology-mediated end joining: A back-up survival mechanism or dedicated pathway. Trends Biochem. Sci. 2015; 40:701–714. 26439531 10.1016/j.tibs.2015.08.006 PMC4638128

[30] Charpentier C., Nora T., Tenaillon O., Clavel F., Hance A.J. Extensive recombination among human immunodeficiency virus type 1 quasispecies makes an important contribution to viral diversity in individual patients. J. Virol. 2006; 80:2472–2482. 16474154 10.1128/JVI.80.5.2472-2482.2006 PMC1395372

[31] Konstantinova P., De Haan P., Das A.T., Berkhout B. Hairpin-induced tRNA-mediated (HITME) recombination in HIV-1. Nucleic Acids Res. 2006; 34:2206–2218. 16670429 10.1093/nar/gkl226 PMC1456326

[32] Onafuwa-Nuga A., Telesnitsky A. The remarkable frequency of human immunodeficiency virus type 1 genetic recombination. Microbiol. Mol. Biol. Rev. 2009; 73:451–480. 19721086 10.1128/MMBR.00012-09 PMC2738136

[33] Maddalo D., Manchado E., Concepcion C.P., Bonetti C., Vidigal J.A., Han Y.C., Ogrodowski P., Crippa A., Rekhtman N., De Stanchina E. et al. In vivo engineering of oncogenic chromosomal rearrangements with the CRISPR/Cas9 system. Nature. 2014; 516:423–427. 25337876 10.1038/nature13902 PMC4270925

[34] Senis E., Fatouros C., Grosse S., Wiedtke E., Niopek D., Mueller A.K., Borner K., Grimm D. CRISPR/Cas9-mediated genome engineering: an adeno-associated viral (AAV) vector toolbox. Biotechnol. J. 2014; 9:1402–1412. 25186301 10.1002/biot.201400046

[35] Binda C.S., Klaver B., Berkhout B., Das A.T. CRISPR-Cas9 dual-gRNA attack causes mutation, excision and inversion of the HIV-1 proviral DNA. Viruses. 2020; 12:330. 10.3390/v12030330 PMC7150824 32197474

[36] Bae S., Kweon J., Kim H.S., Kim J.S. Microhomology-based choice of Cas9 nuclease target sites. Nat. Methods. 2014; 11:705–706. 24972169 10.1038/nmeth.3015

[37] Shen M.W., Arbab M., Hsu J.Y., Worstell D., Culbertson S.J., Krabbe O., Cassa C.A., Liu D.R., Gifford D.K., Sherwood R.I. Predictable and precise template-free CRISPR editing of pathogenic variants. Nature. 2018; 563:646–651. 30405244 10.1038/s41586-018-0686-x PMC6517069

[38] Iyer S., Suresh S., Guo D., Daman K., Chen J.C.J., Liu P., Zieger M., Luk K., Roscoe B.P., Mueller C. et al. Precise therapeutic gene correction by a simple nuclease-induced double-stranded break. Nature. 2019; 568:561–565. 30944467 10.1038/s41586-019-1076-8 PMC6483862

[39] Chang H.H.Y., Pannunzio N.R., Adachi N., Lieber M.R. Non-homologous DNA end joining and alternative pathways to double-strand break repair. Nat. Rev. Mol. Cell Biol. 2017; 18:495–506. 28512351 10.1038/nrm.2017.48 PMC7062608

[40] Chang H.H.Y., Watanabe G., Gerodinnos C.A., Ochi T., Blundell T.L., Jackson S.P., Lieber M.R. Different DNA end configurations dictate which NHEJ components are most important for joining efficiency. J. Biol. Chem. 2016; 291:24377–24389. 27703001 10.1074/jbc.M116.752329 PMC5114395

[41] Ling A.K., So C.C., Le M.X., Chen A.Y., Hung L., Martin A. Double-stranded DNA break polarity skews repair pathway choice during intrachromosomal and interchromosomal recombination. Proc. Natl Acad. Sci. U.S.A. 2018; 115:2800–2805. 29472448 10.1073/pnas.1720962115 PMC5856553

[42] Van Overbeek M., Capurso D., Carter M.M., Thompson M.S., Frias E., Russ C., Reece-Hoyes J.S., Nye C., Gradia S., Vidal B. et al. DNA repair profiling reveals nonrandom outcomes at Cas9-mediated breaks. Mol. Cell. 2016; 63:633–646. 27499295 10.1016/j.molcel.2016.06.037

[43] Chakrabarti A.M., Henser-Brownhill T., Monserrat J., Poetsch A.R., Luscombe N.M., Scaffidi P. Target-specific precision of CRISPR-Mediated genome editing. Mol. Cell. 2019; 73:699–713. 30554945 10.1016/j.molcel.2018.11.031 PMC6395888

[44] Jiang F., Doudna J.A. CRISPR-Cas9 structures and mechanisms. Annu. Rev. Biophys. 2017; 46:505–529. 28375731 10.1146/annurev-biophys-062215-010822

