# Supplementary material for: Extinction of all infectious HIV in cell culture by the CRISPR-Cas12a system with only a single crRNA
Source: Nucleic Acids Res. 2020 Apr 13;48(10):5527–39. doi: 10.1093/nar/gkaa226 (PMC7261156; doi:10.1093/nar/gkaa226)
Supplement: gkaa226_Supplemental_Files [file gkaa226_supplemental_files.zip › NAR-Supplemental Figures.pptx]

## Slide 1
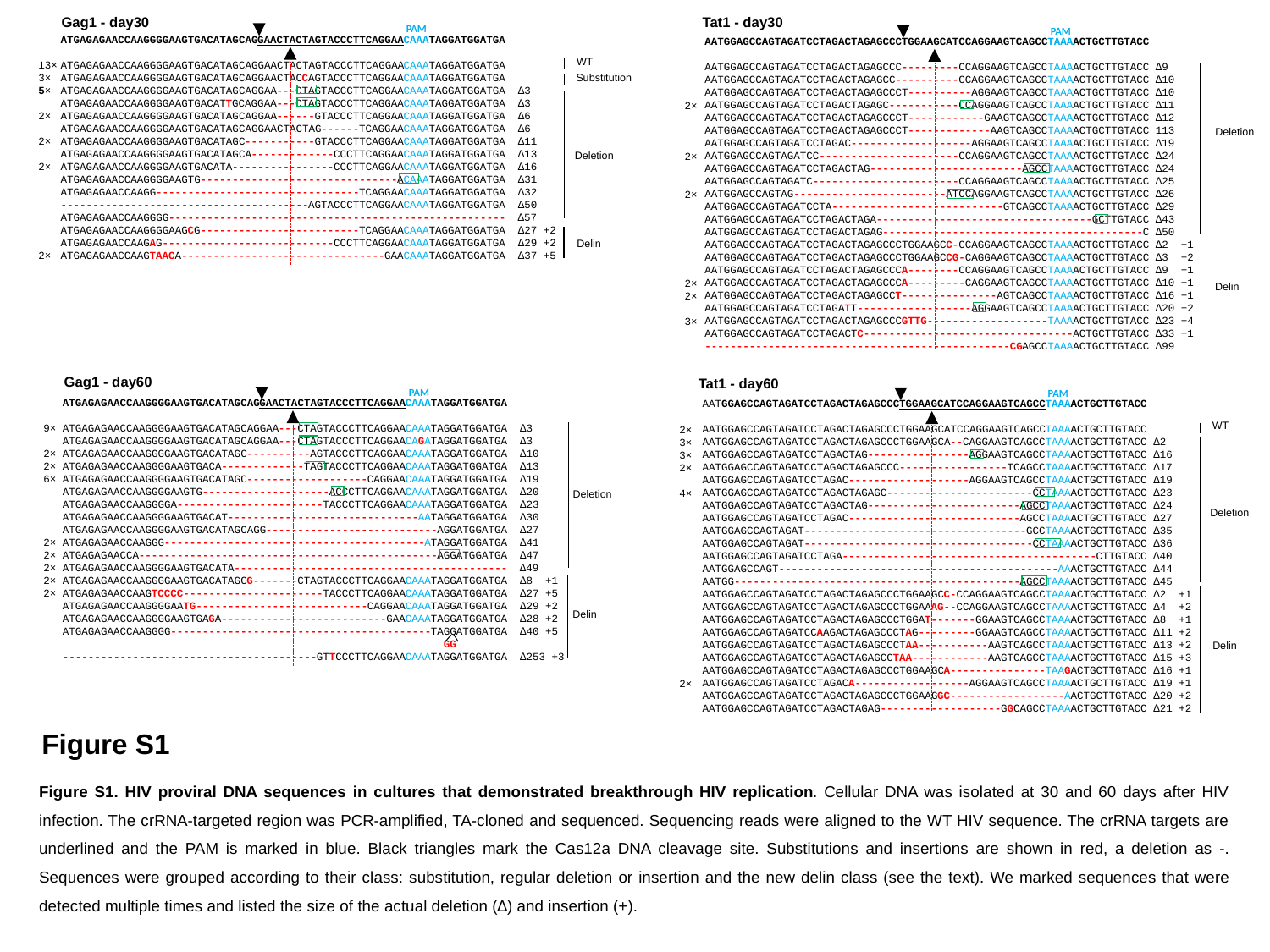

Gag1 - day30
Tat1 - day30
PAM
PAM
ATGAGAGAACCAAGGGGAAGTGACATAGCAGGAACTACTAGTACCCTTCAGGAACAAATAGGATGGATGA
ATGAGAGAACCAAGGGGAAGTGACATAGCAGGAACTACTAGTACCCTTCAGGAACAAATAGGATGGATGA
ATGAGAGAACCAAGGGGAAGTGACATAGCAGGAACTACCAGTACCCTTCAGGAACAAATAGGATGGATGA
ATGAGAGAACCAAGGGGAAGTGACATAGCAGGAA---CTAGTACCCTTCAGGAACAAATAGGATGGATGA ∆3
ATGAGAGAACCAAGGGGAAGTGACATTGCAGGAA---CTAGTACCCTTCAGGAACAAATAGGATGGATGA ∆3
ATGAGAGAACCAAGGGGAAGTGACATAGCAGGAA------GTACCCTTCAGGAACAAATAGGATGGATGA ∆6
ATGAGAGAACCAAGGGGAAGTGACATAGCAGGAACTACTAG------TCAGGAACAAATAGGATGGATGA ∆6
ATGAGAGAACCAAGGGGAAGTGACATAGC-----------GTACCCTTCAGGAACAAATAGGATGGATGA ∆11
ATGAGAGAACCAAGGGGAAGTGACATAGCA-------------CCCTTCAGGAACAAATAGGATGGATGA ∆13 ATGAGAGAACCAAGGGGAAGTGACATA----------------CCCTTCAGGAACAAATAGGATGGATGA ∆16
ATGAGAGAACCAAGGGGAAGTG-------------------------------ACAAATAGGATGGATGA ∆31
ATGAGAGAACCAAGG--------------------------------TCAGGAACAAATAGGATGGATGA ∆32
---------------------------------------AGTACCCTTCAGGAACAAATAGGATGGATGA ∆50
ATGAGAGAACCAAGGGG----------------------------------------------------- ∆57
ATGAGAGAACCAAGGGGAAGCG-------------------------TCAGGAACAAATAGGATGGATGA ∆27 +2
ATGAGAGAACCAAGAG---------------------------CCCTTCAGGAACAAATAGGATGGATGA ∆29 +2 ATGAGAGAACCAAGTAACA--------------------------------GAACAAATAGGATGGATGA ∆37 +5
AATGGAGCCAGTAGATCCTAGACTAGAGCCCTGGAAGCATCCAGGAAGTCAGCCTAAAACTGCTTGTACC
AATGGAGCCAGTAGATCCTAGACTAGAGCCC---------CCAGGAAGTCAGCCTAAAACTGCTTGTACC ∆9
AATGGAGCCAGTAGATCCTAGACTAGAGCC----------CCAGGAAGTCAGCCTAAAACTGCTTGTACC ∆10
AATGGAGCCAGTAGATCCTAGACTAGAGCCCT----------AGGAAGTCAGCCTAAAACTGCTTGTACC ∆10
AATGGAGCCAGTAGATCCTAGACTAGAGC-----------CCAGGAAGTCAGCCTAAAACTGCTTGTACC ∆11 AATGGAGCCAGTAGATCCTAGACTAGAGCCCT------------GAAGTCAGCCTAAAACTGCTTGTACC ∆12
AATGGAGCCAGTAGATCCTAGACTAGAGCCCT-------------AAGTCAGCCTAAAACTGCTTGTACC 113
AATGGAGCCAGTAGATCCTAGAC-------------------AGGAAGTCAGCCTAAAACTGCTTGTACC ∆19
AATGGAGCCAGTAGATCC----------------------CCAGGAAGTCAGCCTAAAACTGCTTGTACC ∆24
AATGGAGCCAGTAGATCCTAGACTAG------------------------AGCCTAAAACTGCTTGTACC ∆24
AATGGAGCCAGTAGATC-----------------------CCAGGAAGTCAGCCTAAAACTGCTTGTACC ∆25
AATGGAGCCAGTAG------------------------ATCCAGGAAGTCAGCCTAAAACTGCTTGTACC ∆26
AATGGAGCCAGTAGATCCTA---------------------------GTCAGCCTAAAACTGCTTGTACC ∆29
AATGGAGCCAGTAGATCCTAGACTAGA----------------------------------GCTTGTACC ∆43
AATGGAGCCAGTAGATCCTAGACTAGAG-----------------------------------------C ∆50
AATGGAGCCAGTAGATCCTAGACTAGAGCCCTGGAAGCC-CCAGGAAGTCAGCCTAAAACTGCTTGTACC ∆2 +1
AATGGAGCCAGTAGATCCTAGACTAGAGCCCTGGAAGCCG-CAGGAAGTCAGCCTAAAACTGCTTGTACC ∆3 +2
AATGGAGCCAGTAGATCCTAGACTAGAGCCCA--------CCAGGAAGTCAGCCTAAAACTGCTTGTACC ∆9 +1
AATGGAGCCAGTAGATCCTAGACTAGAGCCCA---------CAGGAAGTCAGCCTAAAACTGCTTGTACC ∆10 +1
AATGGAGCCAGTAGATCCTAGACTAGAGCCT---------------AGTCAGCCTAAAACTGCTTGTACC ∆16 +1
AATGGAGCCAGTAGATCCTAGATT------------------AGGAAGTCAGCCTAAAACTGCTTGTACC ∆20 +2
AATGGAGCCAGTAGATCCTAGACTAGAGCCCGTTG-------------------TAAAACTGCTTGTACC ∆23 +4
AATGGAGCCAGTAGATCCTAGACTC---------------------------------ACTGCTTGTACC ∆33 +1
------------------------------------------------CGAGCCTAAAACTGCTTGTACC ∆99
WT
13×
3×
5×
2×
2×
2×
2×
Substitution
2×
2×
2×
2×
2×
3×
Deletion
Deletion
Delin
Delin
Gag1 - day60
Tat1 - day60
PAM
PAM
ATGAGAGAACCAAGGGGAAGTGACATAGCAGGAACTACTAGTACCCTTCAGGAACAAATAGGATGGATGA
ATGAGAGAACCAAGGGGAAGTGACATAGCAGGAA---CTAGTACCCTTCAGGAACAAATAGGATGGATGA ∆3
ATGAGAGAACCAAGGGGAAGTGACATAGCAGGAA---CTAGTACCCTTCAGGAACAGATAGGATGGATGA ∆3
ATGAGAGAACCAAGGGGAAGTGACATAGC----------AGTACCCTTCAGGAACAAATAGGATGGATGA ∆10
ATGAGAGAACCAAGGGGAAGTGACA-------------TAGTACCCTTCAGGAACAAATAGGATGGATGA ∆13
ATGAGAGAACCAAGGGGAAGTGACATAGC-------------------CAGGAACAAATAGGATGGATGA ∆19
ATGAGAGAACCAAGGGGAAGTG--------------------ACCCTTCAGGAACAAATAGGATGGATGA ∆20
ATGAGAGAACCAAGGGGA-----------------------TACCCTTCAGGAACAAATAGGATGGATGA ∆23
ATGAGAGAACCAAGGGGAAGTGACAT------------------------------AATAGGATGGATGA ∆30
ATGAGAGAACCAAGGGGAAGTGACATAGCAGG---------------------------AGGATGGATGA ∆27
ATGAGAGAACCAAGGG-----------------------------------------ATAGGATGGATGA ∆41
ATGAGAGAACCA-----------------------------------------------AGGATGGATGA ∆47
ATGAGAGAACCAAGGGGAAGTGACATA------------------------------------------- ∆49
ATGAGAGAACCAAGGGGAAGTGACATAGCG-------CTAGTACCCTTCAGGAACAAATAGGATGGATGA ∆8 +1
ATGAGAGAACCAAGTCCCC----------------------TACCCTTCAGGAACAAATAGGATGGATGA ∆27 +5 ATGAGAGAACCAAGGGGAATG---------------------------CAGGAACAAATAGGATGGATGA ∆29 +2
ATGAGAGAACCAAGGGGAAGTGAGA--------------------------GAACAAATAGGATGGATGA ∆28 +2
ATGAGAGAACCAAGGGG-----------------------------------------TAGGATGGATGA ∆40 +5
 GG
----------------------------------------GTTCCCTTCAGGAACAAATAGGATGGATGA ∆253 +3
AATGGAGCCAGTAGATCCTAGACTAGAGCCCTGGAAGCATCCAGGAAGTCAGCCTAAAACTGCTTGTACC
AATGGAGCCAGTAGATCCTAGACTAGAGCCCTGGAAGCATCCAGGAAGTCAGCCTAAAACTGCTTGTACC
AATGGAGCCAGTAGATCCTAGACTAGAGCCCTGGAAGCA--CAGGAAGTCAGCCTAAAACTGCTTGTACC ∆2 AATGGAGCCAGTAGATCCTAGACTAG----------------AGGAAGTCAGCCTAAAACTGCTTGTACC ∆16
AATGGAGCCAGTAGATCCTAGACTAGAGCCC-----------------TCAGCCTAAAACTGCTTGTACC ∆17
AATGGAGCCAGTAGATCCTAGAC-------------------AGGAAGTCAGCCTAAAACTGCTTGTACC ∆19
AATGGAGCCAGTAGATCCTAGACTAGAGC-----------------------CCTAAAACTGCTTGTACC ∆23
AATGGAGCCAGTAGATCCTAGACTAG------------------------AGCCTAAAACTGCTTGTACC ∆24
AATGGAGCCAGTAGATCCTAGAC---------------------------AGCCTAAAACTGCTTGTACC ∆27
AATGGAGCCAGTAGAT-----------------------------------GCCTAAAACTGCTTGTACC ∆35
AATGGAGCCAGTAGAT------------------------------------CCTAAAACTGCTTGTACC ∆36
AATGGAGCCAGTAGATCCTAGA----------------------------------------CTTGTACC ∆40
AATGGAGCCAGT--------------------------------------------AAACTGCTTGTACC ∆44
AATGG---------------------------------------------AGCCTAAAACTGCTTGTACC ∆45
AATGGAGCCAGTAGATCCTAGACTAGAGCCCTGGAAGCC-CCAGGAAGTCAGCCTAAAACTGCTTGTACC ∆2 +1
AATGGAGCCAGTAGATCCTAGACTAGAGCCCTGGAAAG--CCAGGAAGTCAGCCTAAAACTGCTTGTACC ∆4 +2
AATGGAGCCAGTAGATCCTAGACTAGAGCCCTGGAT-------GGAAGTCAGCCTAAAACTGCTTGTACC ∆8 +1
AATGGAGCCAGTAGATCCAAGACTAGAGCCCTAG---------GGAAGTCAGCCTAAAACTGCTTGTACC ∆11 +2
AATGGAGCCAGTAGATCCTAGACTAGAGCCCTAA-----------AAGTCAGCCTAAAACTGCTTGTACC ∆13 +2
AATGGAGCCAGTAGATCCTAGACTAGAGCCTAA------------AAGTCAGCCTAAAACTGCTTGTACC ∆15 +3
AATGGAGCCAGTAGATCCTAGACTAGAGCCCTGGAAGCA---------------TAAGACTGCTTGTACC ∆16 +1
AATGGAGCCAGTAGATCCTAGACA------------------AGGAAGTCAGCCTAAAACTGCTTGTACC ∆19 +1
AATGGAGCCAGTAGATCCTAGACTAGAGCCCTGGAAGGC------------------AACTGCTTGTACC ∆20 +2
AATGGAGCCAGTAGATCCTAGACTAGAG-------------------GGCAGCCTAAAACTGCTTGTACC ∆21 +2
WT
9×
2×
2×
6×
2×
2×
2×
2×
2×
2×
3×
3×
2×
4×
2×
Deletion
Deletion
Delin
Delin
Figure S1
Figure S1. HIV proviral DNA sequences in cultures that demonstrated breakthrough HIV replication. Cellular DNA was isolated at 30 and 60 days after HIV infection. The crRNA-targeted region was PCR-amplified, TA-cloned and sequenced. Sequencing reads were aligned to the WT HIV sequence. The crRNA targets are underlined and the PAM is marked in blue. Black triangles mark the Cas12a DNA cleavage site. Substitutions and insertions are shown in red, a deletion as -. Sequences were grouped according to their class: substitution, regular deletion or insertion and the new delin class (see the text). We marked sequences that were detected multiple times and listed the size of the actual deletion (∆) and insertion (+).

## Slide 2
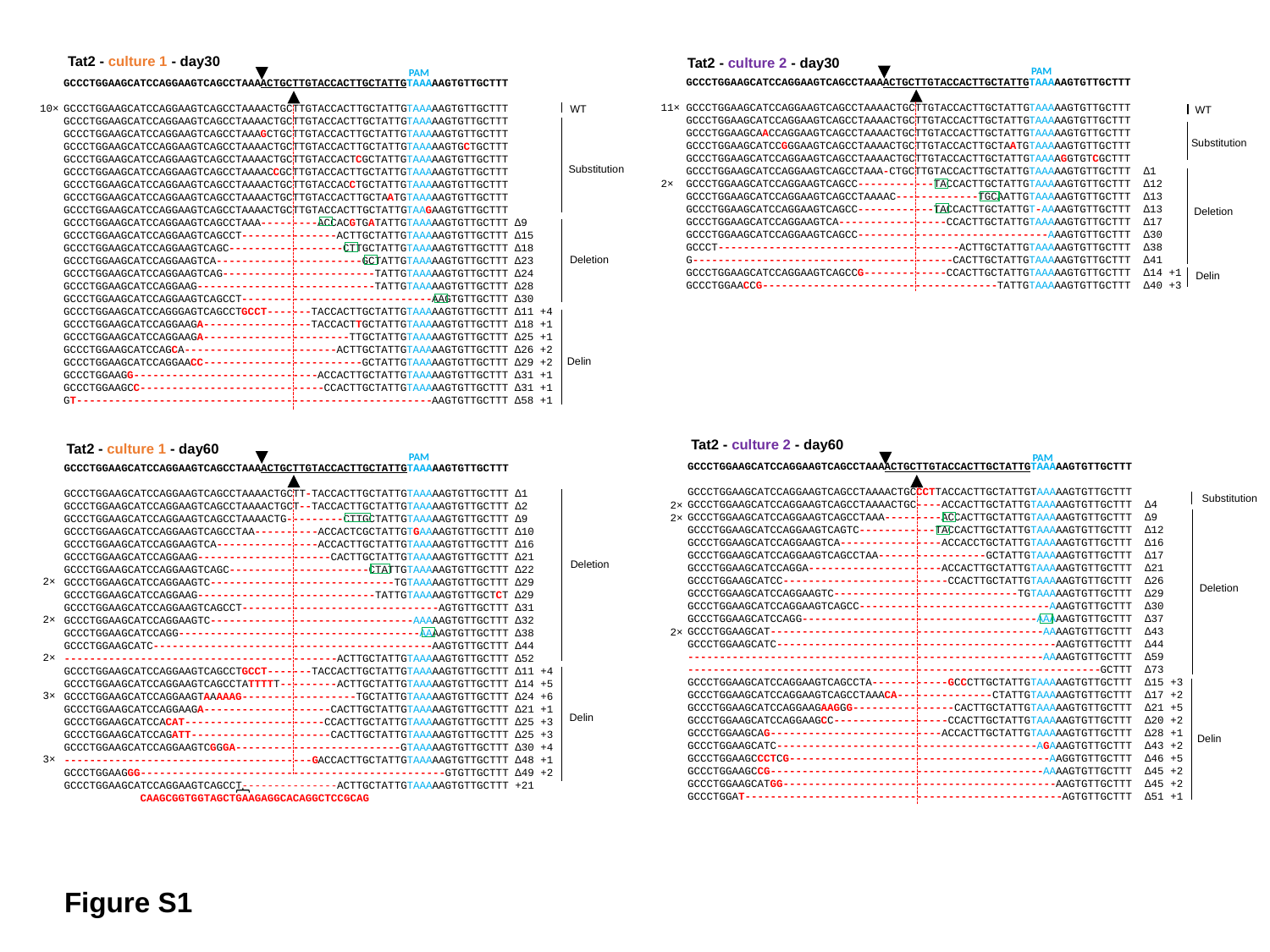

Tat2 - culture 1 - day30
Tat2 - culture 2 - day30
PAM
PAM
GCCCTGGAAGCATCCAGGAAGTCAGCCTAAAACTGCTTGTACCACTTGCTATTGTAAAAAGTGTTGCTTT
GCCCTGGAAGCATCCAGGAAGTCAGCCTAAAACTGCTTGTACCACTTGCTATTGTAAAAAGTGTTGCTTT
GCCCTGGAAGCATCCAGGAAGTCAGCCTAAAACTGCTTGTACCACTTGCTATTGTAAAAAGTGTTGCTTT
GCCCTGGAAGCAACCAGGAAGTCAGCCTAAAACTGCTTGTACCACTTGCTATTGTAAAAAGTGTTGCTTT
GCCCTGGAAGCATCCGGGAAGTCAGCCTAAAACTGCTTGTACCACTTGCTAATGTAAAAAGTGTTGCTTT
GCCCTGGAAGCATCCAGGAAGTCAGCCTAAAACTGCTTGTACCACTTGCTATTGTAAAAGGTGTCGCTTT
GCCCTGGAAGCATCCAGGAAGTCAGCCTAAA-CTGCTTGTACCACTTGCTATTGTAAAAAGTGTTGCTTT ∆1
GCCCTGGAAGCATCCAGGAAGTCAGCC------------TACCACTTGCTATTGTAAAAAGTGTTGCTTT ∆12
GCCCTGGAAGCATCCAGGAAGTCAGCCTAAAAC-------------TGCAATTGTAAAAAGTGTTGCTTT ∆13
GCCCTGGAAGCATCCAGGAAGTCAGCC------------TACCACTTGCTATTGT-AAAAGTGTTGCTTT ∆13
GCCCTGGAAGCATCCAGGAAGTCA-----------------CCACTTGCTATTGTAAAAAGTGTTGCTTT ∆17
GCCCTGGAAGCATCCAGGAAGTCAGCC------------------------------AAAGTGTTGCTTT ∆30
GCCCT--------------------------------------ACTTGCTATTGTAAAAAGTGTTGCTTT ∆38
G-----------------------------------------CACTTGCTATTGTAAAAAGTGTTGCTTT ∆41
GCCCTGGAAGCATCCAGGAAGTCAGCCG-------------CCACTTGCTATTGTAAAAAGTGTTGCTTT ∆14 +1
GCCCTGGAACCG-------------------------------------TATTGTAAAAAGTGTTGCTTT ∆40 +3
GCCCTGGAAGCATCCAGGAAGTCAGCCTAAAACTGCTTGTACCACTTGCTATTGTAAAAAGTGTTGCTTT
GCCCTGGAAGCATCCAGGAAGTCAGCCTAAAACTGCTTGTACCACTTGCTATTGTAAAAAGTGTTGCTTT GCCCTGGAAGCATCCAGGAAGTCAGCCTAAAACTGCTTGTACCACTTGCTATTGTAAAAAGTGTTGCTTT
GCCCTGGAAGCATCCAGGAAGTCAGCCTAAAGCTGCTTGTACCACTTGCTATTGTAAAAAGTGTTGCTTT
GCCCTGGAAGCATCCAGGAAGTCAGCCTAAAACTGCTTGTACCACTTGCTATTGTAAAAAGTGCTGCTTT
GCCCTGGAAGCATCCAGGAAGTCAGCCTAAAACTGCTTGTACCACTCGCTATTGTAAAAAGTGTTGCTTT
GCCCTGGAAGCATCCAGGAAGTCAGCCTAAAACCGCTTGTACCACTTGCTATTGTAAAAAGTGTTGCTTT
GCCCTGGAAGCATCCAGGAAGTCAGCCTAAAACTGCTTGTACCACCTGCTATTGTAAAAAGTGTTGCTTT
GCCCTGGAAGCATCCAGGAAGTCAGCCTAAAACTGCTTGTACCACTTGCTAATGTAAAAAGTGTTGCTTT
GCCCTGGAAGCATCCAGGAAGTCAGCCTAAAACTGCTTGTACCACTTGCTATTGTAAGAAGTGTTGCTTT GCCCTGGAAGCATCCAGGAAGTCAGCCTAAA---------ACCACGTGATATTGTAAAAAGTGTTGCTTT ∆9
GCCCTGGAAGCATCCAGGAAGTCAGCCT---------------ACTTGCTATTGTAAAAAGTGTTGCTTT ∆15
GCCCTGGAAGCATCCAGGAAGTCAGC------------------CTTGCTATTGTAAAAAGTGTTGCTTT ∆18
GCCCTGGAAGCATCCAGGAAGTCA-----------------------GCTATTGTAAAAAGTGTTGCTTT ∆23
GCCCTGGAAGCATCCAGGAAGTCAG------------------------TATTGTAAAAAGTGTTGCTTT ∆24
GCCCTGGAAGCATCCAGGAAG----------------------------TATTGTAAAAAGTGTTGCTTT ∆28
GCCCTGGAAGCATCCAGGAAGTCAGCCT------------------------------AAGTGTTGCTTT ∆30
GCCCTGGAAGCATCCAGGGAGTCAGCCTGCCT-------TACCACTTGCTATTGTAAAAAGTGTTGCTTT ∆11 +4
GCCCTGGAAGCATCCAGGAAGA-----------------TACCACTTGCTATTGTAAAAAGTGTTGCTTT ∆18 +1
GCCCTGGAAGCATCCAGGAAGA-----------------------TTGCTATTGTAAAAAGTGTTGCTTT ∆25 +1
GCCCTGGAAGCATCCAGCA------------------------ACTTGCTATTGTAAAAAGTGTTGCTTT ∆26 +2
GCCCTGGAAGCATCCAGGAACC-------------------------GCTATTGTAAAAAGTGTTGCTTT ∆29 +2
GCCCTGGAAGG-----------------------------ACCACTTGCTATTGTAAAAAGTGTTGCTTT ∆31 +1
GCCCTGGAAGCC-----------------------------CCACTTGCTATTGTAAAAAGTGTTGCTTT ∆31 +1
GT--------------------------------------------------------AAGTGTTGCTTT ∆58 +1
11×
2×
10×
WT
WT
Substitution
Substitution
Deletion
Deletion
Delin
Delin
Tat2 - culture 2 - day60
Tat2 - culture 1 - day60
PAM
PAM
GCCCTGGAAGCATCCAGGAAGTCAGCCTAAAACTGCTTGTACCACTTGCTATTGTAAAAAGTGTTGCTTT
GCCCTGGAAGCATCCAGGAAGTCAGCCTAAAACTGCCCTTACCACTTGCTATTGTAAAAAGTGTTGCTTT
GCCCTGGAAGCATCCAGGAAGTCAGCCTAAAACTGC----ACCACTTGCTATTGTAAAAAGTGTTGCTTT ∆4
GCCCTGGAAGCATCCAGGAAGTCAGCCTAAA---------ACCACTTGCTATTGTAAAAAGTGTTGCTTT ∆9
GCCCTGGAAGCATCCAGGAAGTCAGTC------------TACCACTTGCTATTGTAAAAAGTGTTGCTTT ∆12
GCCCTGGAAGCATCCAGGAAGTCA----------------ACCACCTGCTATTGTAAAAAGTGTTGCTTT ∆16
GCCCTGGAAGCATCCAGGAAGTCAGCCTAA-----------------GCTATTGTAAAAAGTGTTGCTTT ∆17
GCCCTGGAAGCATCCAGGA---------------------ACCACTTGCTATTGTAAAAAGTGTTGCTTT ∆21
GCCCTGGAAGCATCC--------------------------CCACTTGCTATTGTAAAAAGTGTTGCTTT ∆26
GCCCTGGAAGCATCCAGGAAGTC-----------------------------TGTAAAAAGTGTTGCTTT ∆29
GCCCTGGAAGCATCCAGGAAGTCAGCC------------------------------AAAGTGTTGCTTT ∆30
GCCCTGGAAGCATCCAGG-------------------------------------AAAAAGTGTTGCTTT ∆37
GCCCTGGAAGCAT-------------------------------------------AAAAGTGTTGCTTT ∆43
GCCCTGGAAGCATC--------------------------------------------AAGTGTTGCTTT ∆44
--------------------------------------------------------AAAAGTGTTGCTTT ∆59
-----------------------------------------------------------------GCTTT ∆73
GCCCTGGAAGCATCCAGGAAGTCAGCCTA------------GCCCTTGCTATTGTAAAAAGTGTTGCTTT ∆15 +3
GCCCTGGAAGCATCCAGGAAGTCAGCCTAAACA---------------CTATTGTAAAAAGTGTTGCTTT ∆17 +2
GCCCTGGAAGCATCCAGGAAGAAGGG----------------CACTTGCTATTGTAAAAAGTGTTGCTTT ∆21 +5
GCCCTGGAAGCATCCAGGAAGCC------------------CCACTTGCTATTGTAAAAAGTGTTGCTTT ∆20 +2
GCCCTGGAAGCAG---------------------------ACCACTTGCTATTGTAAAAAGTGTTGCTTT ∆28 +1
GCCCTGGAAGCATC-----------------------------------------AGAAAGTGTTGCTTT ∆43 +2
GCCCTGGAAGCCCTCG-----------------------------------------AAGGTGTTGCTTT ∆46 +5
GCCCTGGAAGCCG-------------------------------------------AAAAGTGTTGCTTT ∆45 +2
GCCCTGGAAGCATGG-------------------------------------------AAGTGTTGCTTT ∆45 +2
GCCCTGGAT--------------------------------------------------AGTGTTGCTTT ∆51 +1
GCCCTGGAAGCATCCAGGAAGTCAGCCTAAAACTGCTTGTACCACTTGCTATTGTAAAAAGTGTTGCTTT
GCCCTGGAAGCATCCAGGAAGTCAGCCTAAAACTGCTT-TACCACTTGCTATTGTAAAAAGTGTTGCTTT ∆1
GCCCTGGAAGCATCCAGGAAGTCAGCCTAAAACTGCT--TACCACTTGCTATTGTAAAAAGTGTTGCTTT ∆2
GCCCTGGAAGCATCCAGGAAGTCAGCCTAAAACTG---------CTTGCTATTGTAAAAAGTGTTGCTTT ∆9
GCCCTGGAAGCATCCAGGAAGTCAGCCTAA----------ACCACTCGCTATTGTGAAAAGTGTTGCTTT ∆10
GCCCTGGAAGCATCCAGGAAGTCA----------------ACCACTTGCTATTGTAAAAAGTGTTGCTTT ∆16
GCCCTGGAAGCATCCAGGAAG---------------------CACTTGCTATTGTAAAAAGTGTTGCTTT ∆21
GCCCTGGAAGCATCCAGGAAGTCAGC----------------------CTATTGTAAAAAGTGTTGCTTT ∆22
GCCCTGGAAGCATCCAGGAAGTC-----------------------------TGTAAAAAGTGTTGCTTT ∆29
GCCCTGGAAGCATCCAGGAAG----------------------------TATTGTAAAAAGTGTTGCTCT ∆29
GCCCTGGAAGCATCCAGGAAGTCAGCCT-------------------------------AGTGTTGCTTT ∆31
GCCCTGGAAGCATCCAGGAAGTC--------------------------------AAAAAGTGTTGCTTT ∆32
GCCCTGGAAGCATCCAGG--------------------------------------AAAAGTGTTGCTTT ∆38
GCCCTGGAAGCATC--------------------------------------------AAGTGTTGCTTT ∆44
-------------------------------------------ACTTGCTATTGTAAAAAGTGTTGCTTT ∆52
GCCCTGGAAGCATCCAGGAAGTCAGCCTGCCT-------TACCACTTGCTATTGTAAAAAGTGTTGCTTT ∆11 +4
GCCCTGGAAGCATCCAGGAAGTCAGCCTATTTTT---------ACTTGCTATTGTAAAAAGTGTTGCTTT ∆14 +5
GCCCTGGAAGCATCCAGGAAGTAAAAAG------------------TGCTATTGTAAAAAGTGTTGCTTT ∆24 +6
GCCCTGGAAGCATCCAGGAAGA--------------------CACTTGCTATTGTAAAAAGTGTTGCTTT ∆21 +1
GCCCTGGAAGCATCCACAT----------------------CCACTTGCTATTGTAAAAAGTGTTGCTTT ∆25 +3
GCCCTGGAAGCATCCAGATT----------------------CACTTGCTATTGTAAAAAGTGTTGCTTT ∆25 +3
GCCCTGGAAGCATCCAGGAAGTCGGGA--------------------------GTAAAAAGTGTTGCTTT ∆30 +4
---------------------------------------GACCACTTGCTATTGTAAAAAGTGTTGCTTT ∆48 +1
GCCCTGGAAGGG------------------------------------------------GTGTTGCTTT ∆49 +2
GCCCTGGAAGCATCCAGGAAGTCAGCCT---------------ACTTGCTATTGTAAAAAGTGTTGCTTT +21
 CAAGCGGTGGTAGCTGAAGAGGCACAGGCTCCGCAG
Substitution
2×
2×
2×
Deletion
2×
2×
2×
3×
3×
Deletion
Delin
Delin
Figure S1

## Slide 3
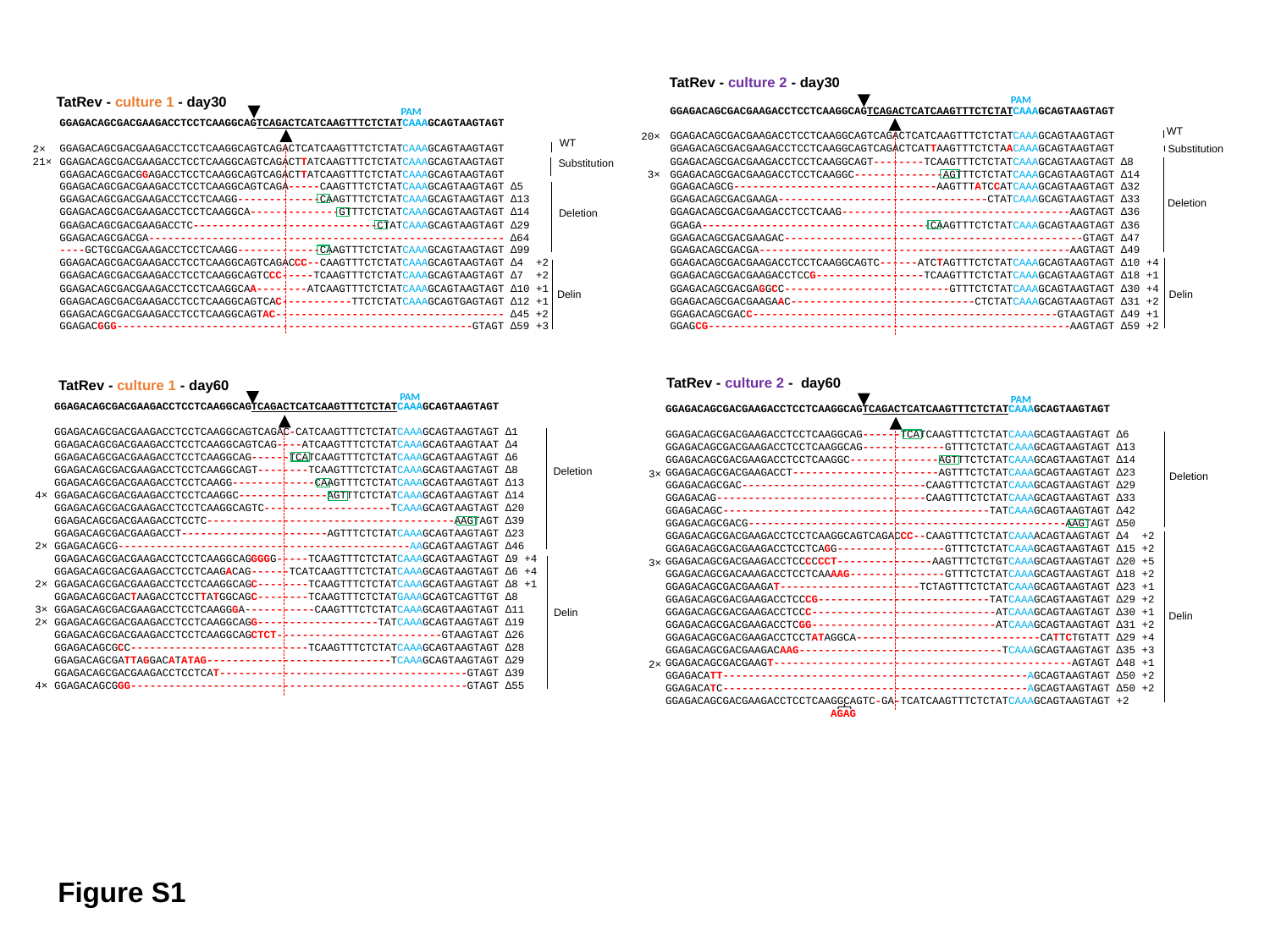

TatRev - culture 2 - day30
TatRev - culture 1 - day30
PAM
GGAGACAGCGACGAAGACCTCCTCAAGGCAGTCAGACTCATCAAGTTTCTCTATCAAAGCAGTAAGTAGT
GGAGACAGCGACGAAGACCTCCTCAAGGCAGTCAGACTCATCAAGTTTCTCTATCAAAGCAGTAAGTAGT
GGAGACAGCGACGAAGACCTCCTCAAGGCAGTCAGACTCATTAAGTTTCTCTAACAAAGCAGTAAGTAGT
GGAGACAGCGACGAAGACCTCCTCAAGGCAGT--------TCAAGTTTCTCTATCAAAGCAGTAAGTAGT ∆8
GGAGACAGCGACGAAGACCTCCTCAAGGC--------------AGTTTCTCTATCAAAGCAGTAAGTAGT ∆14
GGAGACAGCG--------------------------------AAGTTTATCCATCAAAGCAGTAAGTAGT ∆32
GGAGACAGCGACGAAGA---------------------------------CTATCAAAGCAGTAAGTAGT ∆33
GGAGACAGCGACGAAGACCTCCTCAAG------------------------------------AAGTAGT ∆36
GGAGA------------------------------------CAAGTTTCTCTATCAAAGCAGTAAGTAGT ∆36
GGAGACAGCGACGAAGAC-----------------------------------------------GTAGT ∆47
GGAGACAGCGACGA-------------------------------------------------AAGTAGT ∆49
GGAGACAGCGACGAAGACCTCCTCAAGGCAGTC------ATCTAGTTTCTCTATCAAAGCAGTAAGTAGT ∆10 +4
GGAGACAGCGACGAAGACCTCCG-----------------TCAAGTTTCTCTATCAAAGCAGTAAGTAGT ∆18 +1
GGAGACAGCGACGAGGCC--------------------------GTTTCTCTATCAAAGCAGTAAGTAGT ∆30 +4
GGAGACAGCGACGAAGAAC-----------------------------CTCTATCAAAGCAGTAAGTAGT ∆31 +2
GGAGACAGCGACC------------------------------------------------GTAAGTAGT ∆49 +1
GGAGCG---------------------------------------------------------AAGTAGT ∆59 +2
PAM
GGAGACAGCGACGAAGACCTCCTCAAGGCAGTCAGACTCATCAAGTTTCTCTATCAAAGCAGTAAGTAGT
GGAGACAGCGACGAAGACCTCCTCAAGGCAGTCAGACTCATCAAGTTTCTCTATCAAAGCAGTAAGTAGT
GGAGACAGCGACGAAGACCTCCTCAAGGCAGTCAGACTTATCAAGTTTCTCTATCAAAGCAGTAAGTAGT
GGAGACAGCGACGGAGACCTCCTCAAGGCAGTCAGACTTATCAAGTTTCTCTATCAAAGCAGTAAGTAGT
GGAGACAGCGACGAAGACCTCCTCAAGGCAGTCAGA-----CAAGTTTCTCTATCAAAGCAGTAAGTAGT ∆5
GGAGACAGCGACGAAGACCTCCTCAAGG-------------CAAGTTTCTCTATCAAAGCAGTAAGTAGT ∆13
GGAGACAGCGACGAAGACCTCCTCAAGGCA--------------GTTTCTCTATCAAAGCAGTAAGTAGT ∆14
GGAGACAGCGACGAAGACCTC-----------------------------CTATCAAAGCAGTAAGTAGT ∆29
GGAGACAGCGACGA-------------------------------------------------------- ∆64
----GCTGCGACGAAGACCTCCTCAAGG-------------CAAGTTTCTCTATCAAAGCAGTAAGTAGT ∆99
GGAGACAGCGACGAAGACCTCCTCAAGGCAGTCAGACCC--CAAGTTTCTCTATCAAAGCAGTAAGTAGT ∆4 +2
GGAGACAGCGACGAAGACCTCCTCAAGGCAGTCCC-----TCAAGTTTCTCTATCAAAGCAGTAAGTAGT ∆7 +2
GGAGACAGCGACGAAGACCTCCTCAAGGCAA--------ATCAAGTTTCTCTATCAAAGCAGTAAGTAGT ∆10 +1
GGAGACAGCGACGAAGACCTCCTCAAGGCAGTCAC-----------TTCTCTATCAAAGCAGTGAGTAGT ∆12 +1
GGAGACAGCGACGAAGACCTCCTCAAGGCAGTAC------------------------------------ ∆45 +2
GGAGACGGG--------------------------------------------------------GTAGT ∆59 +3
WT
20×
 3×
WT
2×
21×
Substitution
Substitution
Deletion
Deletion
Delin
Delin
TatRev - culture 2 - day60
TatRev - culture 1 - day60
PAM
PAM
GGAGACAGCGACGAAGACCTCCTCAAGGCAGTCAGACTCATCAAGTTTCTCTATCAAAGCAGTAAGTAGT
GGAGACAGCGACGAAGACCTCCTCAAGGCAGTCAGAC-CATCAAGTTTCTCTATCAAAGCAGTAAGTAGT ∆1
GGAGACAGCGACGAAGACCTCCTCAAGGCAGTCAG----ATCAAGTTTCTCTATCAAAGCAGTAAGTAAT ∆4
GGAGACAGCGACGAAGACCTCCTCAAGGCAG------TCATCAAGTTTCTCTATCAAAGCAGTAAGTAGT ∆6
GGAGACAGCGACGAAGACCTCCTCAAGGCAGT--------TCAAGTTTCTCTATCAAAGCAGTAAGTAGT ∆8
GGAGACAGCGACGAAGACCTCCTCAAGG-------------CAAGTTTCTCTATCAAAGCAGTAAGTAGT ∆13
GGAGACAGCGACGAAGACCTCCTCAAGGC--------------AGTTTCTCTATCAAAGCAGTAAGTAGT ∆14
GGAGACAGCGACGAAGACCTCCTCAAGGCAGTC--------------------TCAAAGCAGTAAGTAGT ∆20
GGAGACAGCGACGAAGACCTCCTC---------------------------------------AAGTAGT ∆39
GGAGACAGCGACGAAGACCT-----------------------AGTTTCTCTATCAAAGCAGTAAGTAGT ∆23
GGAGACAGCG----------------------------------------------AAGCAGTAAGTAGT ∆46
GGAGACAGCGACGAAGACCTCCTCAAGGCAGGGGG-----TCAAGTTTCTCTATCAAAGCAGTAAGTAGT ∆9 +4
GGAGACAGCGACGAAGACCTCCTCAAGACAG------TCATCAAGTTTCTCTATCAAAGCAGTAAGTAGT ∆6 +4
GGAGACAGCGACGAAGACCTCCTCAAGGCAGC--------TCAAGTTTCTCTATCAAAGCAGTAAGTAGT ∆8 +1
GGAGACAGCGACTAAGACCTCCTTATGGCAGC--------TCAAGTTTCTCTATGAAAGCAGTCAGTTGT ∆8
GGAGACAGCGACGAAGACCTCCTCAAGGGA-----------CAAGTTTCTCTATCAAAGCAGTAAGTAGT ∆11
GGAGACAGCGACGAAGACCTCCTCAAGGCAGG-------------------TATCAAAGCAGTAAGTAGT ∆19
GGAGACAGCGACGAAGACCTCCTCAAGGCAGCTCT--------------------------GTAAGTAGT ∆26
GGAGACAGCGCC----------------------------TCAAGTTTCTCTATCAAAGCAGTAAGTAGT ∆28
GGAGACAGCGATTAGGACATATAG-----------------------------TCAAAGCAGTAAGTAGT ∆29
GGAGACAGCGACGAAGACCTCCTCAT---------------------------------------GTAGT ∆39
GGAGACAGCGGG-----------------------------------------------------GTAGT ∆55
GGAGACAGCGACGAAGACCTCCTCAAGGCAGTCAGACTCATCAAGTTTCTCTATCAAAGCAGTAAGTAGT
GGAGACAGCGACGAAGACCTCCTCAAGGCAG------TCATCAAGTTTCTCTATCAAAGCAGTAAGTAGT ∆6
GGAGACAGCGACGAAGACCTCCTCAAGGCAG-------------GTTTCTCTATCAAAGCAGTAAGTAGT ∆13
GGAGACAGCGACGAAGACCTCCTCAAGGC--------------AGTTTCTCTATCAAAGCAGTAAGTAGT ∆14
GGAGACAGCGACGAAGACCT-----------------------AGTTTCTCTATCAAAGCAGTAAGTAGT ∆23
GGAGACAGCGAC-----------------------------CAAGTTTCTCTATCAAAGCAGTAAGTAGT ∆29
GGAGACAG---------------------------------CAAGTTTCTCTATCAAAGCAGTAAGTAGT ∆33
GGAGACAGC------------------------------------------TATCAAAGCAGTAAGTAGT ∆42
GGAGACAGCGACG--------------------------------------------------AAGTAGT ∆50
GGAGACAGCGACGAAGACCTCCTCAAGGCAGTCAGACCC--CAAGTTTCTCTATCAAAACAGTAAGTAGT ∆4 +2
GGAGACAGCGACGAAGACCTCCTCAGG-----------------GTTTCTCTATCAAAGCAGTAAGTAGT ∆15 +2
GGAGACAGCGACGAAGACCTCCCCCCT---------------AAGTTTCTCTGTCAAAGCAGTAAGTAGT ∆20 +5
GGAGACAGCGACAAAGACCTCCTCAAAAG---------------GTTTCTCTATCAAAGCAGTAAGTAGT ∆18 +2
GGAGACAGCGACGAAGAT----------------------TCTAGTTTCTCTATCAAAGCAGTAAGTAGT ∆23 +1
GGAGACAGCGACGAAGACCTCCCG---------------------------TATCAAAGCAGTAAGTAGT ∆29 +2
GGAGACAGCGACGAAGACCTCCC-----------------------------ATCAAAGCAGTAAGTAGT ∆30 +1
GGAGACAGCGACGAAGACCTCGG-----------------------------ATCAAAGCAGTAAGTAGT ∆31 +2
GGAGACAGCGACGAAGACCTCCTATAGGCA-----------------------------CATTCTGTATT ∆29 +4
GGAGACAGCGACGAAGACAAG--------------------------------TCAAAGCAGTAAGTAGT ∆35 +3
GGAGACAGCGACGAAGT-----------------------------------------------AGTAGT ∆48 +1
GGAGACATT------------------------------------------------AGCAGTAAGTAGT ∆50 +2
GGAGACATC------------------------------------------------AGCAGTAAGTAGT ∆50 +2
GGAGACAGCGACGAAGACCTCCTCAAGGCAGTC-GA-TCATCAAGTTTCTCTATCAAAGCAGTAAGTAGT +2
 AGAG
Deletion
3×
3×
2×
Deletion
4×
2×
2×
3×
2×
4×
Delin
Delin
Figure S1

## Slide 4
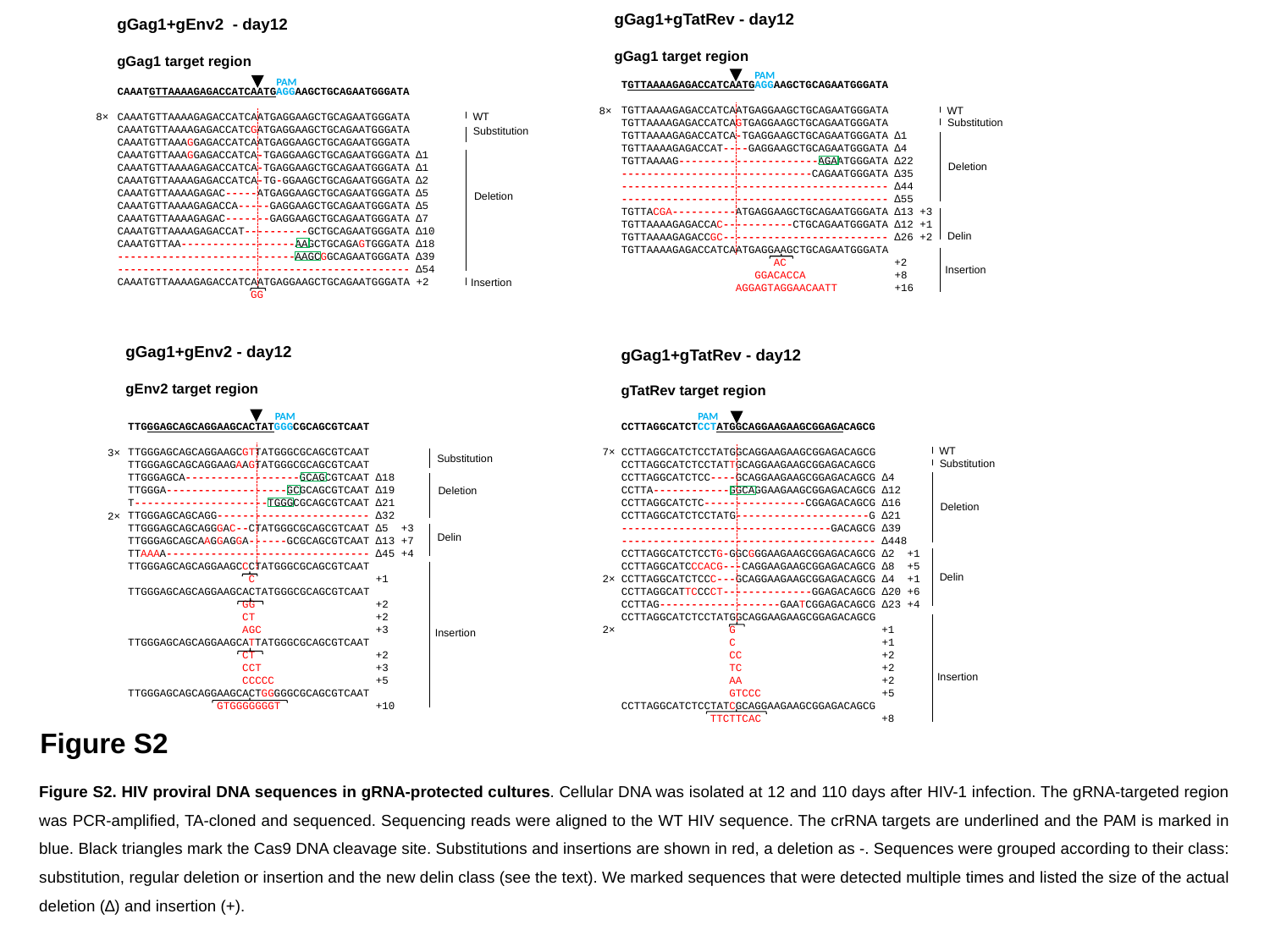

gGag1+gTatRev - day12
gGag1 target region
gGag1+gEnv2 - day12
gGag1 target region
PAM
PAM
TGTTAAAAGAGACCATCAATGAGGAAGCTGCAGAATGGGATA
TGTTAAAAGAGACCATCAATGAGGAAGCTGCAGAATGGGATA
TGTTAAAAGAGACCATCAGTGAGGAAGCTGCAGAATGGGATA
TGTTAAAAGAGACCATCA-TGAGGAAGCTGCAGAATGGGATA ∆1
TGTTAAAAGAGACCAT----GAGGAAGCTGCAGAATGGGATA ∆4
TGTTAAAAG----------------------AGAATGGGATA ∆22
------------------------------CAGAATGGGATA ∆35
------------------------------------------ ∆44
------------------------------------------ ∆55
TGTTACGA----------ATGAGGAAGCTGCAGAATGGGATA ∆13 +3
TGTTAAAAGAGACCAC-----------CTGCAGAATGGGATA ∆12 +1
TGTTAAAAGAGACCGC-------------------------- ∆26 +2
TGTTAAAAGAGACCATCAATGAGGAAGCTGCAGAATGGGATA
 AC +2
 GGACACCA +8
 AGGAGTAGGAACAATT +16
CAAATGTTAAAAGAGACCATCAATGAGGAAGCTGCAGAATGGGATA
CAAATGTTAAAAGAGACCATCAATGAGGAAGCTGCAGAATGGGATA
CAAATGTTAAAAGAGACCATCGATGAGGAAGCTGCAGAATGGGATA
CAAATGTTAAAGGAGACCATCAATGAGGAAGCTGCAGAATGGGATA
CAAATGTTAAAGGAGACCATCA-TGAGGAAGCTGCAGAATGGGATA ∆1
CAAATGTTAAAAGAGACCATCA-TGAGGAAGCTGCAGAATGGGATA ∆1
CAAATGTTAAAAGAGACCATCA-TG-GGAAGCTGCAGAATGGGATA ∆2
CAAATGTTAAAAGAGAC-----ATGAGGAAGCTGCAGAATGGGATA ∆5
CAAATGTTAAAAGAGACCA-----GAGGAAGCTGCAGAATGGGATA ∆5
CAAATGTTAAAAGAGAC-------GAGGAAGCTGCAGAATGGGATA ∆7
CAAATGTTAAAAGAGACCAT----------GCTGCAGAATGGGATA ∆10
CAAATGTTAA------------------AAGCTGCAGAGTGGGATA ∆18
----------------------------AAGCGGCAGAATGGGATA ∆39
---------------------------------------------- ∆54
CAAATGTTAAAAGAGACCATCAATGAGGAAGCTGCAGAATGGGATA +2
 GG
WT
8×
8×
WT
Substitution
Substitution
Deletion
Deletion
Delin
Insertion
Insertion
gGag1+gEnv2 - day12
gEnv2 target region
gGag1+gTatRev - day12
gTatRev target region
CCTTAGGCATCTCCTATGGCAGGAAGAAGCGGAGACAGCG
CCTTAGGCATCTCCTATGGCAGGAAGAAGCGGAGACAGCG
CCTTAGGCATCTCCTATTGCAGGAAGAAGCGGAGACAGCG
CCTTAGGCATCTCC----GCAGGAAGAAGCGGAGACAGCG ∆4
CCTTA------------GGCAGGAAGAAGCGGAGACAGCG ∆12
CCTTAGGCATCTC----------------CGGAGACAGCG ∆16
CCTTAGGCATCTCCTATG---------------------G ∆21
---------------------------------GACAGCG ∆39
---------------------------------------- ∆448
CCTTAGGCATCTCCTG-GGCGGGAAGAAGCGGAGACAGCG ∆2 +1
CCTTAGGCATCCCACG---CAGGAAGAAGCGGAGACAGCG ∆8 +5
CCTTAGGCATCTCCC---GCAGGAAGAAGCGGAGACAGCG ∆4 +1
CCTTAGGCATTCCCCT--------------GGAGACAGCG ∆20 +6
CCTTAG-------------------GAATCGGAGACAGCG ∆23 +4
CCTTAGGCATCTCCTATGGCAGGAAGAAGCGGAGACAGCG
 G +1
 C +1
 CC +2
 TC +2
 AA +2
 GTCCC +5
CCTTAGGCATCTCCTATCGCAGGAAGAAGCGGAGACAGCG
 TTCTTCAC +8
PAM
PAM
TTGGGAGCAGCAGGAAGCACTATGGGCGCAGCGTCAAT
TTGGGAGCAGCAGGAAGCGTTATGGGCGCAGCGTCAAT
TTGGGAGCAGCAGGAAGAAGTATGGGCGCAGCGTCAAT
TTGGGAGCA------------------GCAGCGTCAAT ∆18
TTGGGA-------------------GCGCAGCGTCAAT ∆19
T---------------------TGGGCGCAGCGTCAAT ∆21
TTGGGAGCAGCAGG------------------------ ∆32
TTGGGAGCAGCAGGGAC--CTATGGGCGCAGCGTCAAT ∆5 +3
TTGGGAGCAGCAAGGAGGA------GCGCAGCGTCAAT ∆13 +7
TTAAAA-------------------------------- ∆45 +4
TTGGGAGCAGCAGGAAGCCCTATGGGCGCAGCGTCAAT
 C +1
TTGGGAGCAGCAGGAAGCACTATGGGCGCAGCGTCAAT
 GG +2
 CT +2
 AGC +3
TTGGGAGCAGCAGGAAGCATTATGGGCGCAGCGTCAAT
 CT +2
 CCT +3
 CCCCC +5
TTGGGAGCAGCAGGAAGCACTGGGGGCGCAGCGTCAAT
 GTGGGGGGGT +10
WT
7×
2×
2×
3×
2×
Substitution
Substitution
Deletion
Deletion
Delin
Delin
Insertion
Insertion
Figure S2
Figure S2. HIV proviral DNA sequences in gRNA-protected cultures. Cellular DNA was isolated at 12 and 110 days after HIV-1 infection. The gRNA-targeted region was PCR-amplified, TA-cloned and sequenced. Sequencing reads were aligned to the WT HIV sequence. The crRNA targets are underlined and the PAM is marked in blue. Black triangles mark the Cas9 DNA cleavage site. Substitutions and insertions are shown in red, a deletion as -. Sequences were grouped according to their class: substitution, regular deletion or insertion and the new delin class (see the text). We marked sequences that were detected multiple times and listed the size of the actual deletion (∆) and insertion (+).

## Slide 5
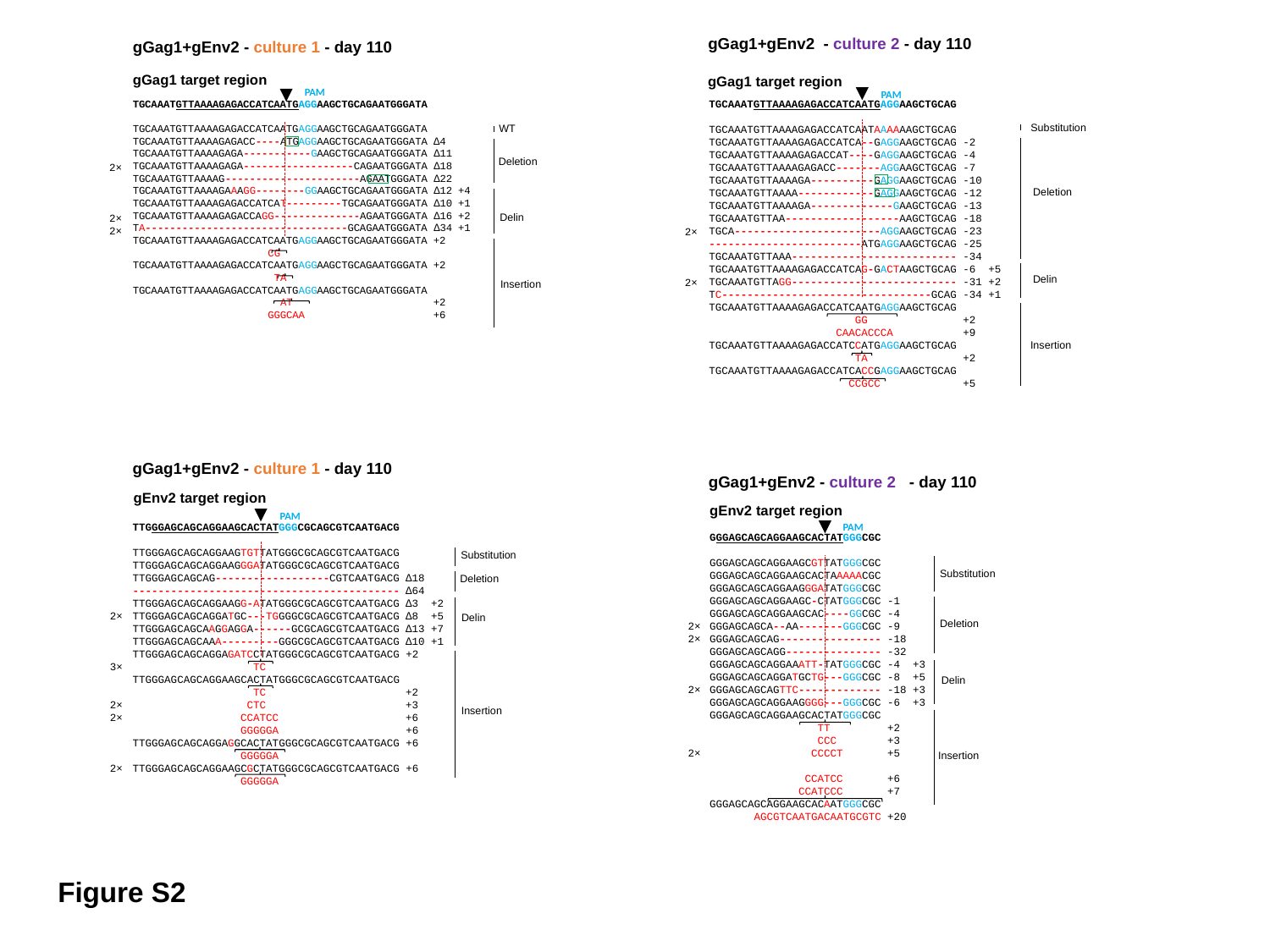

gGag1+gEnv2 - culture 2 - day 110
gGag1+gEnv2 - culture 1 - day 110
gGag1 target region
gGag1 target region
PAM
PAM
TGCAAATGTTAAAAGAGACCATCAATGAGGAAGCTGCAGAATGGGATA
TGCAAATGTTAAAAGAGACCATCAATGAGGAAGCTGCAGAATGGGATA
TGCAAATGTTAAAAGAGACC----ATGAGGAAGCTGCAGAATGGGATA ∆4
TGCAAATGTTAAAAGAGA-----------GAAGCTGCAGAATGGGATA ∆11
TGCAAATGTTAAAAGAGA------------------CAGAATGGGATA ∆18
TGCAAATGTTAAAAG----------------------AGAATGGGATA ∆22
TGCAAATGTTAAAAGAAAGG--------GGAAGCTGCAGAATGGGATA ∆12 +4
TGCAAATGTTAAAAGAGACCATCAT---------TGCAGAATGGGATA ∆10 +1
TGCAAATGTTAAAAGAGACCAGG--------------AGAATGGGATA ∆16 +2
TA---------------------------------GCAGAATGGGATA ∆34 +1
TGCAAATGTTAAAAGAGACCATCAATGAGGAAGCTGCAGAATGGGATA +2
 CG TGCAAATGTTAAAAGAGACCATCAATGAGGAAGCTGCAGAATGGGATA +2
 TA TGCAAATGTTAAAAGAGACCATCAATGAGGAAGCTGCAGAATGGGATA
 AT +2
 GGGCAA +6
TGCAAATGTTAAAAGAGACCATCAATGAGGAAGCTGCAG
TGCAAATGTTAAAAGAGACCATCAATAAAAAAGCTGCAG
TGCAAATGTTAAAAGAGACCATCA--GAGGAAGCTGCAG -2
TGCAAATGTTAAAAGAGACCAT----GAGGAAGCTGCAG -4
TGCAAATGTTAAAAGAGACC-------AGGAAGCTGCAG -7
TGCAAATGTTAAAAGA----------GAGGAAGCTGCAG -10
TGCAAATGTTAAAA------------GAGGAAGCTGCAG -12
TGCAAATGTTAAAAGA-------------GAAGCTGCAG -13
TGCAAATGTTAA------------------AAGCTGCAG -18
TGCA-----------------------AGGAAGCTGCAG -23
------------------------ATGAGGAAGCTGCAG -25
TGCAAATGTTAAA-------------------------- -34
TGCAAATGTTAAAAGAGACCATCAG-GACTAAGCTGCAG -6 +5
TGCAAATGTTAGG-------------------------- -31 +2
TC---------------------------------GCAG -34 +1
TGCAAATGTTAAAAGAGACCATCAATGAGGAAGCTGCAG
 GG +2
 CAACACCCA +9
TGCAAATGTTAAAAGAGACCATCCATGAGGAAGCTGCAG
 TA +2
TGCAAATGTTAAAAGAGACCATCACCGAGGAAGCTGCAG
 CCGCC +5
Substitution
WT
2×
2×
2×
2×
2×
Deletion
Deletion
Delin
Delin
Insertion
Insertion
gGag1+gEnv2 - culture 1 - day 110
gGag1+gEnv2 - culture 2 - day 110
gEnv2 target region
gEnv2 target region
PAM
TTGGGAGCAGCAGGAAGCACTATGGGCGCAGCGTCAATGACG
TTGGGAGCAGCAGGAAGTGTTATGGGCGCAGCGTCAATGACG
TTGGGAGCAGCAGGAAGGGATATGGGCGCAGCGTCAATGACG
TTGGGAGCAGCAG------------------CGTCAATGACG ∆18
------------------------------------------ ∆64
TTGGGAGCAGCAGGAAGG-ATATGGGCGCAGCGTCAATGACG ∆3 +2
TTGGGAGCAGCAGGATGC---TGGGGCGCAGCGTCAATGACG ∆8 +5
TTGGGAGCAGCAAGGAGGA------GCGCAGCGTCAATGACG ∆13 +7
TTGGGAGCAGCAAA---------GGGCGCAGCGTCAATGACG ∆10 +1
TTGGGAGCAGCAGGAGATCCTATGGGCGCAGCGTCAATGACG +2
 TC TTGGGAGCAGCAGGAAGCACTATGGGCGCAGCGTCAATGACG
 TC +2
 CTC +3
 CCATCC +6
 GGGGGA +6
TTGGGAGCAGCAGGAGGCACTATGGGCGCAGCGTCAATGACG +6
 GGGGGA
TTGGGAGCAGCAGGAAGCGCTATGGGCGCAGCGTCAATGACG +6
 GGGGGA
PAM
GGGAGCAGCAGGAAGCACTATGGGCGC
GGGAGCAGCAGGAAGCGTTATGGGCGC
GGGAGCAGCAGGAAGCACTAAAAACGC GGGAGCAGCAGGAAGGGATATGGGCGC
GGGAGCAGCAGGAAGC-CTATGGGCGC -1
GGGAGCAGCAGGAAGCAC----GGCGC -4
GGGAGCAGCA--AA-------GGGCGC -9
GGGAGCAGCAG---------------- -18
GGGAGCAGCAGG--------------- -32
GGGAGCAGCAGGAAATT-TATGGGCGC -4 +3
GGGAGCAGCAGGATGCTG---GGGCGC -8 +5
GGGAGCAGCAGTTC------------- -18 +3
GGGAGCAGCAGGAAGGGG---GGGCGC -6 +3
GGGAGCAGCAGGAAGCACTATGGGCGC
 TT +2
 CCC +3
 CCCCT +5
 CCATCC +6
 CCATCCC +7
GGGAGCAGCAGGAAGCACAATGGGCGC
 AGCGTCAATGACAATGCGTC +20
2×
3×
2×
2×
2×
Substitution
Substitution
Deletion
2×
2×
2×
2×
Delin
Deletion
Delin
Insertion
Insertion
Figure S2

## Slide 6
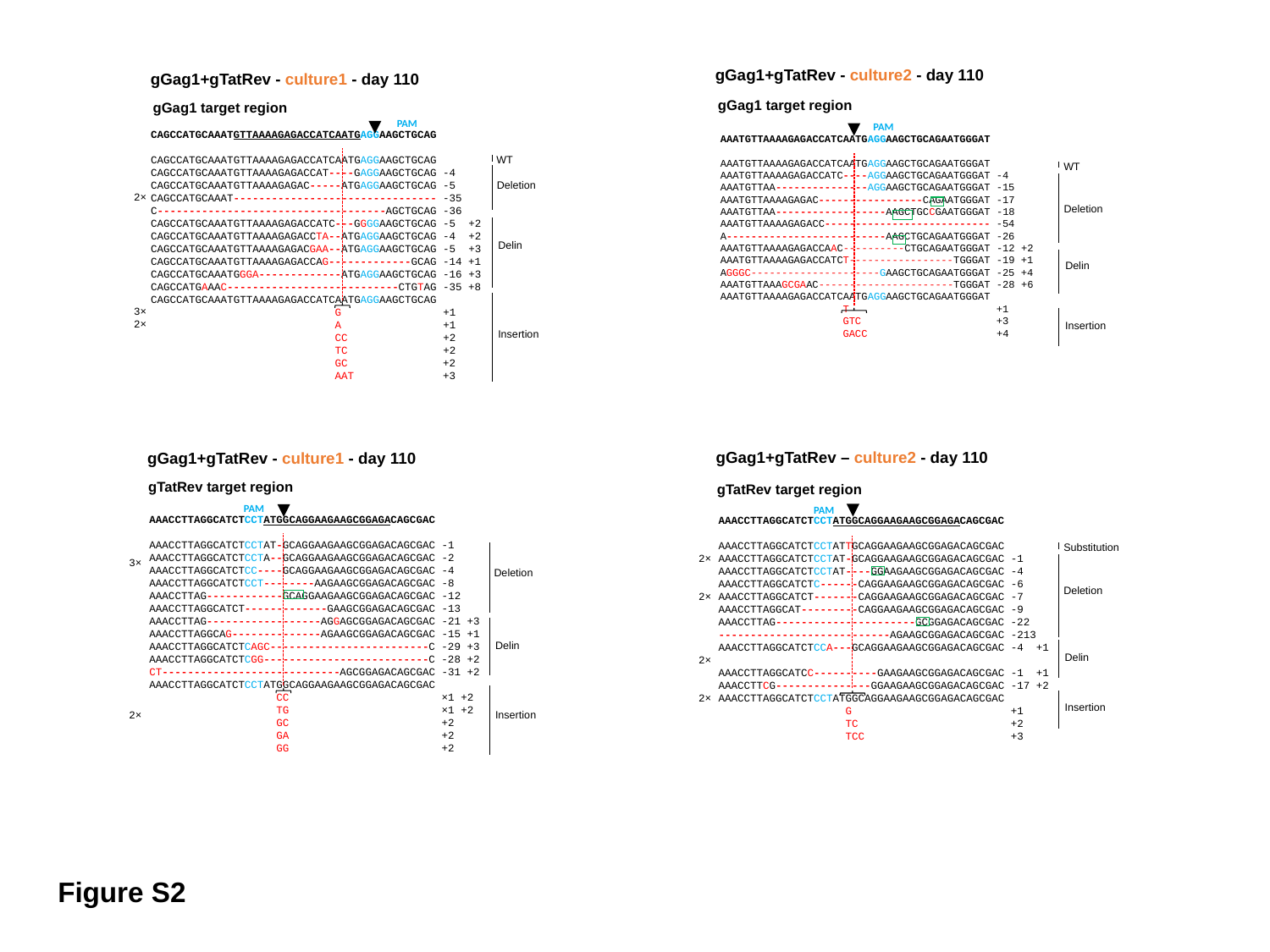

gGag1+gTatRev - culture2 - day 110
gGag1+gTatRev - culture1 - day 110
gGag1 target region
gGag1 target region
PAM
PAM
CAGCCATGCAAATGTTAAAAGAGACCATCAATGAGGAAGCTGCAG
CAGCCATGCAAATGTTAAAAGAGACCATCAATGAGGAAGCTGCAG
CAGCCATGCAAATGTTAAAAGAGACCAT----GAGGAAGCTGCAG -4
CAGCCATGCAAATGTTAAAAGAGAC-----ATGAGGAAGCTGCAG -5
CAGCCATGCAAAT-------------------------------- -35
C------------------------------------AGCTGCAG -36
CAGCCATGCAAATGTTAAAAGAGACCATC---GGGGAAGCTGCAG -5 +2
CAGCCATGCAAATGTTAAAAGAGACCTA--ATGAGGAAGCTGCAG -4 +2
CAGCCATGCAAATGTTAAAAGAGACGAA--ATGAGGAAGCTGCAG -5 +3
CAGCCATGCAAATGTTAAAAGAGACCAG-------------GCAG -14 +1
CAGCCATGCAAATGGGA-------------ATGAGGAAGCTGCAG -16 +3
CAGCCATGAAAC---------------------------CTGTAG -35 +8
CAGCCATGCAAATGTTAAAAGAGACCATCAATGAGGAAGCTGCAG
 G +1
 A +1
 CC +2
 TC +2
 GC +2
 AAT +3
AAATGTTAAAAGAGACCATCAATGAGGAAGCTGCAGAATGGGAT
AAATGTTAAAAGAGACCATCAATGAGGAAGCTGCAGAATGGGAT
AAATGTTAAAAGAGACCATC----AGGAAGCTGCAGAATGGGAT -4
AAATGTTAA---------------AGGAAGCTGCAGAATGGGAT -15
AAATGTTAAAAGAGAC-----------------CAGAATGGGAT -17
AAATGTTAA------------------AAGCTGCCGAATGGGAT -18
AAATGTTAAAAGAGACC--------------------------- -54
A--------------------------AAGCTGCAGAATGGGAT -26
AAATGTTAAAAGAGACCAAC----------CTGCAGAATGGGAT -12 +2
AAATGTTAAAAGAGACCATCT-----------------TGGGAT -19 +1
AGGGC---------------------GAAGCTGCAGAATGGGAT -25 +4
AAATGTTAAAGCGAAC----------------------TGGGAT -28 +6
AAATGTTAAAAGAGACCATCAATGAGGAAGCTGCAGAATGGGAT
 T +1
 GTC +3
 GACC +4
WT
WT
2×
3×
2×
Deletion
Deletion
Delin
Delin
Insertion
Insertion
gGag1+gTatRev – culture2 - day 110
gGag1+gTatRev - culture1 - day 110
gTatRev target region
gTatRev target region
AAACCTTAGGCATCTCCTATGGCAGGAAGAAGCGGAGACAGCGAC
AAACCTTAGGCATCTCCTATTGCAGGAAGAAGCGGAGACAGCGAC
AAACCTTAGGCATCTCCTAT-GCAGGAAGAAGCGGAGACAGCGAC -1
AAACCTTAGGCATCTCCTAT----GGAAGAAGCGGAGACAGCGAC -4
AAACCTTAGGCATCTC------CAGGAAGAAGCGGAGACAGCGAC -6
AAACCTTAGGCATCT-------CAGGAAGAAGCGGAGACAGCGAC -7
AAACCTTAGGCAT---------CAGGAAGAAGCGGAGACAGCGAC -9
AAACCTTAG----------------------GCGGAGACAGCGAC -22
---------------------------AGAAGCGGAGACAGCGAC -213
AAACCTTAGGCATCTCCA---GCAGGAAGAAGCGGAGACAGCGAC -4 +1
AAACCTTAGGCATCC----------GAAGAAGCGGAGACAGCGAC -1 +1
AAACCTTCG---------------GGAAGAAGCGGAGACAGCGAC -17 +2
AAACCTTAGGCATCTCCTATGGCAGGAAGAAGCGGAGACAGCGAC
 G +1
 TC +2
 TCC +3
PAM
PAM
AAACCTTAGGCATCTCCTATGGCAGGAAGAAGCGGAGACAGCGAC
AAACCTTAGGCATCTCCTAT-GCAGGAAGAAGCGGAGACAGCGAC -1
AAACCTTAGGCATCTCCTA--GCAGGAAGAAGCGGAGACAGCGAC -2
AAACCTTAGGCATCTCC----GCAGGAAGAAGCGGAGACAGCGAC -4
AAACCTTAGGCATCTCCT--------AAGAAGCGGAGACAGCGAC -8
AAACCTTAG------------GCAGGAAGAAGCGGAGACAGCGAC -12
AAACCTTAGGCATCT-------------GAAGCGGAGACAGCGAC -13
AAACCTTAG------------------AGGAGCGGAGACAGCGAC -21 +3
AAACCTTAGGCAG--------------AGAAGCGGAGACAGCGAC -15 +1
AAACCTTAGGCATCTCAGC-------------------------C -29 +3
AAACCTTAGGCATCTCGG--------------------------C -28 +2
CT----------------------------AGCGGAGACAGCGAC -31 +2
AAACCTTAGGCATCTCCTATGGCAGGAAGAAGCGGAGACAGCGAC
 CC ×1 +2
 TG ×1 +2
 GC +2
 GA +2
 GG +2
2×
2×
2×
2×
Substitution
3×
2×
Deletion
Deletion
Delin
Delin
Insertion
Insertion
Figure S2
