# Supplementary material for: Extinction of all infectious HIV in cell culture by the CRISPR-Cas12a system with only a single crRNA
Source: Nucleic Acids Res. 2020 Apr 13;48(10):5527–39. doi: 10.1093/nar/gkaa226 (PMC7261156; doi:10.1093/nar/gkaa226)
Supplement: gkaa226_Supplemental_Files [file gkaa226_supplemental_files.zip › NAR-Supplemental Tables.docx]

**Table S1. crRNAs targeting HIV-1**

| **crRNA** | **position in HIV-1 LAI DNA** | **Orientation** | **PAM** | **Target sequence (5-3)** | **Specificity score^A^** | **Shannon Entropy^B^** |
| --- | --- | --- | --- | --- | --- | --- |
| LTR1 | 130-152, 9296-9284 | sense | TTTG | GATGGTGCTACAAGCTAGTACCA | 97 | 0.20 |
| LTR2 | 159-181, 9291-9313 | anti-sense | TTTA | TTGGCCTCTTCTACCTTATCTGG | 88 | 0.44 |
| LTR3 | 268-290, 9400-9422 | sense | TTTG | ACAGCCGCCTAGCATTTCATCAC | 96 | 0.40 |
| LTR4 | 286-308, 9418-9400 | sense | TTTC | ATCACGTGGCCCGAGAGCTGCAT | 98 | 0.38 |
| LTR5 | 359-381, 9491-9513 | sense | TTTC | CGCTGGGGACTTTCCAGGGAGGC | 96 | 0.36 |
| LTR6 | 373-395, 9505-9527 | sense | TTTC | CAGGGAGGCGTGGCCTGGGCGGG | 97 | 0.53 |
| Gag1 | 1502-1524 | anti-sense | TTTG | TTCCTGAAGGGTACTAGTAGTTC | 97 | 0.14 |
| Gag2 | 1317-1339 | anti-sense | TTTA | AATCTTGTGGGGTGGCTCCTTCT | 97 | 0.10 |
| GagPol1 | 2284-2306 | sense | TTTA | ACTTCCCTCAGATCACTCTTTGG | 91 | 0.16 |
| GagPol2 | 2306-2328 | sense | TTTG | GCAACGACCCCTCGTCACAATAA | 100 | 0.21 |
| Pol1 | 2861-2883 | anti-sense | TTTA | ACCCTGCGGGATGTGGTATTCCT | 98 | 0.16 |
| Pol2 | 3656-3678 | anti-sense | TTTA | CATCATTAGTGTGGGCACCCCTC | 98 | 0.22 |
| Pol3 | 4629-4651 | sense | TTTC | ACCAGTACTACGGTTAAGGCCGC | 99 | 0.30 |
| Vpr1 | 5698-5720 | sense | TTTC | CTAGGATTTGGCTCCATGGCTTA | 95 | 0.35 |
| Vpr2 | 5708-5730 | sense | TTTG | GCTCCATGGCTTAGGGCAACATA | 98 | 0.30 |
| Tat1 | 5896-5918 | anti-sense | TTTA | GGCTGACTTCCTGGATGCTTCCA | 95 | 0.10 |
| Tat2 | 5923-5945 | anti-sense | TTTA | CAATAGCAAGTGGTACAAGCAGT | 96 | 0.24 |
| TatRev | 6051-6073 | anti-sense | TTTG | ATAGAGAAACTTGATGAGTCTGA | 90 | 0.40 |
| Env1 | 6684-6706 | anti-sense | TTTC | CCCGCTACTACTATTGGTATTAC | 99 | 0.59 |
| Env2 | 6925-6947 | anti-sense | TTTA | GAATCGCAAAACCAGCCGGGGCA | 100 | 0.19 |
| Env3 | 7572-7594 | anti-sense | TTTG | TCCGCTGATGGGAGGGGCATACA | 98 | 0.19 |
| Nef1 | 8872-8894 | anti-sense | TTTC | CCTTACAGTAGGCCATCCAACCA | 97 | 0.60 |
| Nef2 | 8932-8954 | anti-sense | TTTC | CAGGTCTCGAGATGCTGCTCCCA | 96 | 0.18 |

**A**. The crRNA specificity score was calculated with guide RNA design software from Benchling. The specificity score ranges from 0 to 100 and a high score means high specificity.

**B**. The Shannon entropy was calculated to estimate the variation in the gRNA target sequence amongst virus isolates (group M) present in the HIV database 2014 (hiv.lanl.gov; only complete HIV genomes were included). The entropy can vary from 0 to 1.5, with an invariant sequence having a score of 0.

**Table S2. Primers used for amplification and sequencing of crRNA target regions in HIV**

| **Primer name** | **Oligonucleotide Sequence (5’-3’)** | **Orientation** | **Target Region** |
| --- | --- | --- | --- |
| 5LTR F | AACGAAGACAAGATATCCTTGA | Forward | 5LTR |
| 5LTR R | TTAATACTGACGCTCTCGCA | Reverse |  |
| 3LTR F | CAGCATCTCGAGACCTGGAAAAACAT | Forward | 3LTR |
| 3LTR R | CGGGCACACACTACTTGAAGC | Reverse |  |
| Gag1 F | CATATCACCTAGAACTTTAAATGC | Forward | Gag |
| Gag1 R | AGTTTTATAGAACCGGTCTACATA | Reverse |  |
| TatRev F | AACTTATGGGGATACTTGGG | Forward | Tat/Rev |
| TatRev R | CTATGATTACTATGGACCACACA | Reverse |  |
| T7 | TAATACGACTCACTATAGGG | Forward | Top-cloning vector |
| M13RP | CAGGAAACAGCTATGAC | Reverse |  |

| **System** | **n** | **Deletion** | | | **Insertion** | | | **Delin** | | |  |
| --- | --- | --- | --- | --- | --- | --- | --- | --- | --- | --- | --- |
|  |  | n | % | Size (bp) | n | % | Size (bp) | n | % | Size (bp) | **Ref.** |
| Mammalian | 10 | 10 | 100 | 2-24 | - |  |  | - |  |  | (1) |
|  | 9 | 9 | 100 | 3-11 | - |  |  | - |  |  | (2) |
|  | 2 | 2 | 100 | 5, 7 | - |  |  | - |  |  | (3) |
|  | 10 | 10 | 100 | 20-110 | - |  |  | - |  |  | (4) |
|  | 60 | 60 | 100 | 4-87 | - |  |  | - |  |  | (5) |
|  | 36 | 36 | 100 | 3-26 | - |  |  | - |  |  | (6) |
|  | 13 | 10 | 76.9 | 4-39 | - |  |  | 3 | 23.1 | 10-34 | (7) |
|  | 20 | 20 | 100 | 2-16 | - |  |  | - |  |  | (8) |
|  | 6 | 6 | 100 | 3-10 | - |  |  | - |  |  | (9) |
|  | 50 | 45 | 90.0 | 4-31 | - |  |  | 5 | 10.0 | 1-15 | (10) |
|  | 21 | 19 | 90.5 | 3-58 | - |  |  | 2 | 9.5 | 10,21 | (11) |
|  | 8 | 7 | 87.5 | 1-14 | 1 | 12.5 | 1 | - |  |  | (12) |
|  | 20 | 20 | 100 | 1-13 | - |  |  | - |  |  | (13) |
|  | 44 | 40 | 90.9 | 1-1809 | - |  |  | 4 | 9.1 | 11-23 | (14) |
|  | 2 | 1 | 50.0 | 24 | 1 | 50.0 | 1 | - |  |  | (15) |
|  | 8 | 8 | 100 | 6-38 | - |  |  | - |  |  | (16) |
|  | 6 | 3 | 50.0 | 1-20 | - |  |  | 3 | 50.0 | 1-4 | (17) |
|  | 2 | 2 | 100 | 10, 11 | - |  |  | - |  |  | (18) |
|  | 27 | 27 | 100 | 1-20 | - |  |  | - |  |  | (19) |
|  | 19 | 15 | 78.9 | 2-15 | - |  |  | 4 | 21.1 | 10-15 | (20) |
|  | 73 | 40 | 54.8 | 14-49 | - |  |  | 33 | 45.2 | 4-37 | (21) |
|  | 33 | 27 | 81.8 | 2-227 | 2 | 6.1 | 11, 33 | 4 | 12.1 | 5-21 | (22) |
|  | 5 | 5 | 100 | 3-21 | - |  |  | - |  |  | (23) |
|  | 34 | 29 | 85.3 | 1-201 | - |  |  | 5 | 14.7 | 3-10 | (24) |
|  | 5 | 5 | 100 | 4-22 | - |  |  | - |  |  | (25) |
| Non-mammalian | 29 | 27 | 93.1 | 4-22 | - |  |  | 2 | 6.9 | 2, 4 | (26) |
|  | 15 | 14 | 93.3 | 7-17 | - |  |  | 1 | 6.7 | 4 | (25) |
| Plant | 65 | 56 | 86.2 | 1-43 | - |  |  | 9 | 13.8 | 6-43 | (27) |
|  | 52 | 49 | 94.2 | 3-39 | - |  |  | 3 | 5.8 | 9-41 | (28) |
|  | 47 | 47 | 100 | 3-28 | - |  |  | - |  |  | (29) |
|  | 11 | 10 | 90.9 | 6-73 | 1 | 9.1 | 1 | - |  |  | (30) |
|  | 22 | 22 | 100 | 4-32 | - |  |  | - |  |  | (31) |
|  | 12 | 12 | 100 | 6-22 | - |  |  | - |  |  | (32) |
|  | 38 | 35 | 92.1 | 1-39 | 2 | 5.3 | 3 | 1 | 2.6 | 13 | (33) |
|  | 40 | 38 | 95.0 | 1-16 | 2 | 5.0 | 1 | - |  |  | (34) |
|  | 10 | 9 | 90.0 | 1-22 | - |  |  | 1 | 10.0 | 61 | (35) |
|  | 38 | 37 | 97.3 | 3-106 | - |  |  | 1 | 2.6 | 6 | (36) |
|  | 47 | 41 | 87.2 | 3-32 | - |  |  | 6 | 12.8 | 1-28 | (37) |
|  | 19 | 18 | 94.7 | 7-287 | - |  |  | 1 | 5.3 | 16 | (38) |
|  | 27 | 27 | 100 | 4-38 | - |  |  | - |  |  | (39) |
|  | 8 | 8 | 100 | 6-38 | - |  |  | - |  |  | (40) |
|  | 26 | 26 | 100 | 3-28 | - |  |  | - |  |  | (41) |
|  | 10 | 8 | 80.0 | 1-13 | - |  |  | 2 | 20.0 | 7, 15 | (42) |
|  | 12 | 12 | 100 | 3-75 | - |  |  | - |  |  | (43) |
|  | 2 | 2 | 100 | 13, 14 | - |  |  | - |  |  | (44) |
|  | 72 | 67 | 93.1 | 3-30 | 1 | 1.4 | 1 | 4 | 5.5 | 3-92 | (45) |
|  | 9 | 9 | 100 | 2-11 | - |  |  | - |  |  | (46) |
|  | 17 | 17 | 100 | 1-20 | - |  |  | - |  |  | (47) |
| Bacteria | 4 | 4 | 100 | 50-7500 | - |  |  | - |  |  | (48) |
|  | 7 | 7 | 100 | 409-1624 | - |  |  | - |  |  | (49) |
|  | 165 | 159 | 96.4 | 2-1530 | - |  |  | 6 | 3.6 | 3-129 | (50) |
|  | 14 | 13 | 92.9 | 2-14 | 1 | 7.1 | 2 | - |  |  | (51) |
|  | 6 | 6 | 100 | ∼1000 | - |  |  | - |  |  | (52) |
|  | 22 | 20 | 90.9 | 1-80 | - |  |  | 2 | 9.1 | 2-8 | (53) |
|  | 197 | 175 | 88.8 | 4-57 | - |  |  | 22 | 11.2 | 5-47 | (54) |
| Cell free | 3 | 3 | 100 | 7-102 | - |  |  | - |  |  | (55) |

**Table S3. Literature survey of Cas12a-edited DNA sequences**

**References**

1. Zetsche, B., Gootenberg, J.S., Abudayyeh, O.O., Slaymaker, I.M., Makarova, K.S., Essletzbichler, P., Volz, S.E., Joung, J., van der Oost, J., Regev, A. *et al.* (2015) Cpf1 is a single RNA-guided endonuclease of a class 2 CRISPR-Cas system. *Cell*, **163**, 759-771.

2. Zetsche, B., Heidenreich, M., Mohanraju, P., Fedorova, I., Kneppers, J., DeGennaro, E.M., Winblad, N., Choudhury, S.R., Abudayyeh, O.O., Gootenberg, J.S. *et al.* (2017) Multiplex gene editing by CRISPR-Cpf1 using a single crRNA array. *Nat Biotechnol*, **35**, 31-34.

3. Breinig, M., Schweitzer, A.Y., Herianto, A.M., Revia, S., Schaefer, L., Wendler, L., Cobos Galvez, A. and Tschaharganeh, D.F. (2019) Multiplexed orthogonal genome editing and transcriptional activation by Cas12a. *Nat Methods*, **16**, 51-54.

4. Campa, C.C., Weisbach, N.R., Santinha, A.J., Incarnato, D. and Platt, R.J. (2019) Multiplexed genome engineering by Cas12a and CRISPR arrays encoded on single transcripts. *Nat Methods*, **16**, 887-893.

5. Li, B., Zhao, W., Luo, X., Zhang, X., Li, C., Zeng, C. and Dong, Y. (2017) Engineering CRISPR-Cpf1 crRNAs and mRNAs to maximize genome editing efficiency. *Nat Biomed Eng*, **1**.

6. Zhang, Y., Long, C., Li, H., McAnally, J.R., Baskin, K.K., Shelton, J.M., Bassel-Duby, R. and Olson, E.N. (2017) CRISPR-Cpf1 correction of muscular dystrophy mutations in human cardiomyocytes and mice. *Science advances*, **3**, e1602814.

7. Kim, Y., Cheong, S.A., Lee, J.G., Lee, S.W., Lee, M.S., Baek, I.J. and Sung, Y.H. (2016) Generation of knockout mice by Cpf1-mediated gene targeting. *Nat Biotechnol*, **34**, 808-810.

8. Kim, D., Kim, J., Hur, J.K., Been, K.W., Yoon, S.H. and Kim, J.S. (2016) Genome-wide analysis reveals specificities of Cpf1 endonucleases in human cells. *Nat Biotechnol*, **34**, 863-868.

9. Gao, L., Cox, D.B.T., Yan, W.X., Manteiga, J.C., Schneider, M.W., Yamano, T., Nishimasu, H., Nureki, O., Crosetto, N. and Zhang, F. (2017) Engineered Cpf1 variants with altered PAM specificities. *Nat Biotechnol*, **35**, 789-792.

10. Hur, J.K., Kim, K., Been, K.W., Baek, G., Ye, S., Hur, J.W., Ryu, S.M., Lee, Y.S. and Kim, J.S. (2016) Targeted mutagenesis in mice by electroporation of Cpf1 ribonucleoproteins. *Nat Biotechnol*, **34**, 807-808.

11. Tu, M., Lin, L., Cheng, Y., He, X., Sun, H., Xie, H., Fu, J., Liu, C., Li, J., Chen, D. *et al.* (2017) A 'new lease of life': FnCpf1 possesses DNA cleavage activity for genome editing in human cells. *Nucleic Acids Res*, **45**, 11295-11304.

12. Nihongaki, Y., Otabe, T., Ueda, Y. and Sato, M. (2019) A split CRISPR-Cpf1 platform for inducible genome editing and gene activation. *Nat Chem Biol*, **15**, 882-888.

13. Chow, R.D., Wang, G., Ye, L., Codina, A., Kim, H.R., Shen, L., Dong, M.B., Errami, Y. and Chen, S. (2019) In vivo profiling of metastatic double knockouts through CRISPR-Cpf1 screens. *Nat Methods*, **16**, 405-408.

14. Wu, H., Liu, Q., Shi, H., Xie, J., Zhang, Q., Ouyang, Z., Li, N., Yang, Y., Liu, Z., Zhao, Y. *et al.* (2018) Engineering CRISPR/Cpf1 with tRNA promotes genome editing capability in mammalian systems. *Cellular and molecular life sciences : CMLS*, **75**, 3593-3607.

15. Zhou, M., Hu, Z., Qiu, L., Zhou, T., Feng, M., Hu, Q., Zeng, B., Li, Z., Sun, Q., Wu, Y. *et al.* (2018) Seamless genetic conversion of SMN2 to SMN1 via CRISPR/Cpf1 and single-stranded oligodeoxynucleotides in spinal muscular atrophy patient-specific induced pluripotent stem cells. *Human gene therapy*, **29**, 1252-1263.

16. Lee, J.G., Ha, C.H., Yoon, B., Cheong, S.A., Kim, G., Lee, D.J., Woo, D.C., Kim, Y.H., Nam, S.Y., Lee, S.W. *et al.* (2019) Knockout rat models mimicking human atherosclerosis created by Cpf1-mediated gene targeting. *Sci Rep*, **9**, 2628.

17. Ahn, W.C., Park, K.H., Bak, I.S., Song, H.N., An, Y., Lee, S.J., Jung, M., Yoo, K.W., Yu, D.Y., Kim, Y.S. *et al.* (2019) In vivo genome editing using the Cpf1 ortholog derived from Eubacterium eligens. *Sci Rep*, **9**, 13911.

18. Ma, X., Chen, X., Jin, Y., Ge, W., Wang, W., Kong, L., Ji, J., Guo, X., Huang, J., Feng, X.H. *et al.* (2018) Small molecules promote CRISPR-Cpf1-mediated genome editing in human pluripotent stem cells. *Nat Commun*, **9**, 1303.

19. Chow, R.D., Kim, H.R. and Chen, S. (2018) Programmable sequential mutagenesis by inducible Cpf1 crRNA array inversion. *Nat Commun*, **9**, 1903.

20. Yang, M., Wei, H., Wang, Y., Deng, J., Tang, Y., Zhou, L., Guo, G. and Tong, A. (2017) Targeted disruption of V600E-mutant BRAF gene by CRISPR-Cpf1. *Mol Ther Nucleic Acids*, **8**, 450-458.

21. Watkins-Chow, D.E., Varshney, G.K., Garrett, L.J., Chen, Z., Jimenez, E.A., Rivas, C., Bishop, K.S., Sood, R., Harper, U.L., Pavan, W.J. *et al.* (2017) Highly efficient Cpf1-mediated gene targeting in mice following high concentration pronuclear injection. *G3 (Bethesda, Md.)*, **7**, 719-722.

22. Sun, H., Li, F., Liu, J., Yang, F., Zeng, Z., Lv, X., Tu, M., Liu, Y., Ge, X., Liu, C. *et al.* (2018) A single multiplex crRNA array for FnCpf1-mediated human genome editing. *Mol Ther*, **26**, 2070-2076.

23. Kim, Y.S., Kim, G.R., Park, M., Yang, S.C., Park, S.H., Won, J.E., Lee, J.H., Shin, H.E., Song, H. and Kim, H.R. (2020) Electroporation of AsCpf1/RNP at the zygote stage is an efficient genome editing method to generate knock-out mice deficient in leukemia inhibitory factor. *Tissue engineering and regenerative medicine*, **17**, 45-53.

24. Li, P., Zhang, L., Li, Z., Xu, C., Du, X. and Wu, S. (2019) Cas12a mediates efficient and precise endogenous gene tagging via MITI: microhomology-dependent targeted integrations. *Cellular and molecular life sciences : CMLS*.

25. Wierson, W.A., Simone, B.W., WareJoncas, Z., Mann, C., Welker, J.M., Kar, B., Emch, M.J., Friedberg, I., Gendron, W.A.C., Barry, M.A. *et al.* (2019) Expanding the CRISPR toolbox with ErCas12a in zebrafish and human cells. *The CRISPR journal*, **2**, 417-433.

26. Moreno-Mateos, M.A., Fernandez, J.P., Rouet, R., Vejnar, C.E., Lane, M.A., Mis, E., Khokha, M.K., Doudna, J.A. and Giraldez, A.J. (2017) CRISPR-Cpf1 mediates efficient homology-directed repair and temperature-controlled genome editing. *Nat Commun*, **8**, 2024.

27. Endo, A., Masafumi, M., Kaya, H. and Toki, S. (2016) Efficient targeted mutagenesis of rice and tobacco genomes using Cpf1 from Francisella novicida. *Sci Rep*, **6**.

28. Malzahn, A.A., Tang, X., Lee, K., Ren, Q., Sretenovic, S., Zhang, Y., Chen, H., Kang, M., Bao, Y., Zheng, X. *et al.* (2019) Application of CRISPR-Cas12a temperature sensitivity for improved genome editing in rice, maize, and Arabidopsis. *BMC biology*, **17**, 9.

29. Li, B., Rui, H., Li, Y., Wang, Q., Alariqi, M., Qin, L., Sun, L., Ding, X., Wang, F., Zou, J. *et al.* (2019) Robust CRISPR/Cpf1 (Cas12a)-mediated genome editing in allotetraploid cotton (Gossypium hirsutum). *Plant biotechnology journal*, **17**, 1862-1864.

30. Lee, K., Zhang, Y., Kleinstiver, B.P., Guo, J.A., Aryee, M.J., Miller, J., Malzahn, A., Zarecor, S., Lawrence-Dill, C.J., Joung, J.K. *et al.* (2019) Activities and specificities of CRISPR/Cas9 and Cas12a nucleases for targeted mutagenesis in maize. *Plant biotechnology journal*, **17**, 362-372.

31. Tang, X., Lowder, L.G., Zhang, T., Malzahn, A.A., Zheng, X., Voytas, D.F., Zhong, Z., Chen, Y., Ren, Q., Li, Q. *et al.* (2017) A CRISPR-Cpf1 system for efficient genome editing and transcriptional repression in plants. *Nature plants*, **3**, 17018.

32. Xu, R., Qin, R., Li, H., Li, J., Yang, J. and Wei, P. (2019) Enhanced genome editing in rice using single transcript unit CRISPR-LbCpf1 systems. *Plant biotechnology journal*, **17**, 553-555.

33. Zhong, Z., Zhang, Y., You, Q., Tang, X., Ren, Q., Liu, S., Yang, L., Wang, Y., Liu, X., Liu, B. *et al.* (2018) Plant genome editing using FnCpf1 and LbCpf1 nucleases at redefined and altered PAM sites. *Molecular plant*, **11**, 999-1002.

34. Kim, H., Kim, S.T., Ryu, J., Kang, B.C., Kim, J.S. and Kim, S.G. (2017) CRISPR/Cpf1-mediated DNA-free plant genome editing. *Nat Commun*, **8**, 14406.

35. Yin, X., Biswal, A.K., Dionora, J., Perdigon, K.M., Balahadia, C.P., Mazumdar, S., Chater, C., Lin, H.C., Coe, R.A., Kretzschmar, T. *et al.* (2017) CRISPR-Cas9 and CRISPR-Cpf1 mediated targeting of a stomatal developmental gene EPFL9 in rice. *Plant cell reports*, **36**, 745-757.

36. Wang, M., Mao, Y., Lu, Y., Tao, X. and Zhu, J.K. (2017) Multiplex gene editing in rice using the CRISPR-Cpf1 system. *Molecular plant*, **10**, 1011-1013.

37. Xu, R., Qin, R., Li, H., Li, D., Li, L., Wei, P. and Yang, J. (2017) Generation of targeted mutant rice using a CRISPR-Cpf1 system. *Plant biotechnology journal*, **15**, 713-717.

38. Li, S., Zhang, X., Wang, W., Guo, X., Wu, Z., Du, W., Zhao, Y. and Xia, L. (2018) Expanding the scope of CRISPR/Cpf1-mediated genome editing in rice. *Molecular plant*, **11**, 995-998.

39. Tang, X., Liu, G., Zhou, J., Ren, Q., You, Q., Tian, L., Xin, X., Zhong, Z., Liu, B., Zheng, X. *et al.* (2018) A large-scale whole-genome sequencing analysis reveals highly specific genome editing by both Cas9 and Cpf1 (Cas12a) nucleases in rice. *Genome Biol*, **19**, 84.

40. Hu, X., Wang, C., Liu, Q., Fu, Y. and Wang, K. (2017) Targeted mutagenesis in rice using CRISPR-Cpf1 system. *Journal of genetics and genomics = Yi chuan xue bao*, **44**, 71-73.

41. Wang, M., Mao, Y., Lu, Y., Wang, Z., Tao, X. and Zhu, J.K. (2018) Multiplex gene editing in rice with simplified CRISPR-Cpf1 and CRISPR-Cas9 systems. *Journal of integrative plant biology*, **60**, 626-631.

42. Jia, H., Orbovic, V. and Wang, N. (2019) CRISPR-LbCas12a-mediated modification of citrus. *Plant biotechnology journal*, **17**, 1928-1937.

43. Begemann, M.B., Gray, B.N., January, E., Gordon, G.C., He, Y., Liu, H., Wu, X., Brutnell, T.P., Mockler, T.C. and Oufattole, M. (2017) Precise insertion and guided editing of higher plant genomes using Cpf1 CRISPR nucleases. *Sci Rep*, **7**, 11606.

44. Schindele, P. and Puchta, H. (2019) Engineering CRISPR/LbCas12a for highly efficient, temperature-tolerant plant gene editing. *Plant biotechnology journal*.

45. Hsu, C.T., Cheng, Y.J., Yuan, Y.H., Hung, W.F., Cheng, Q.W., Wu, F.H., Lee, L.Y., Gelvin, S.B. and Lin, C.S. (2019) Application of Cas12a and nCas9-activation-induced cytidine deaminase for genome editing and as a non-sexual strategy to generate homozygous/multiplex edited plants in the allotetraploid genome of tobacco. *Plant molecular biology*, **101**, 355-371.

46. Li, S., Zhang, Y., Xia, L. and Qi, Y. (2019) CRISPR-Cas12a enables efficient biallelic gene targeting in rice. *Plant biotechnology journal*.

47. Banakar, R., Schubert, M., Collingwood, M., Vakulskas, C., Eggenberger, A.L. and Wang, K. (2020) Comparison of CRISPR-Cas9/Cas12a ribonucleoprotein complexes for genome editing efficiency in the rice phytoene desaturase (OsPDS) gene. *Rice (New York, N.Y.)*, **13**, 4.

48. Jiang, Y., Qian, F., Yang, J., Liu, Y., Dong, F., Xu, C., Sun, B., Chen, B., Xu, X., Li, Y. *et al.* (2017) CRISPR-Cpf1 assisted genome editing of Corynebacterium glutamicum. *Nat Commun*, **8**, 15179.

49. Li, L., Wei, K., Zheng, G., Liu, X., Chen, S., Jiang, W. and Lu, Y. (2018) CRISPR-Cpf1-assisted multiplex genome editing and transcriptional repression in Streptomyces. *Applied and environmental microbiology*, **84**.

50. Sun, B., Yang, J., Yang, S., Ye, R.D., Chen, D. and Jiang, Y. (2018) A CRISPR-Cpf1-assisted non-homologous end joining genome editing system of Mycobacterium smegmatis. *Biotechnol J*, **13**, e1700588.

51. Yang, Z., Edwards, H. and Xu, P. (2020) CRISPR-Cas12a/Cpf1-assisted precise, efficient and multiplexed genome-editing in Yarrowia lipolytica. *Metabolic engineering communications*, **10**, e00112.

52. Li, Z.H., Liu, M., Lyu, X.M., Wang, F.Q. and Wei, D.Z. (2018) CRISPR/Cpf1 facilitated large fragment deletion in Saccharomyces cerevisiae. *Journal of basic microbiology*, **58**, 1100-1104.

53. Ferenczi, A., Pyott, D.E., Xipnitou, A. and Molnar, A. (2017) Efficient targeted DNA editing and replacement in Chlamydomonas reinhardtii using Cpf1 ribonucleoproteins and single-stranded DNA. *Proc Natl Acad Sci U S A*, **114**, 13567-13572.

54. Pu, X., Liu, L., Li, P., Huo, H., Dong, X., Xie, K., Yang, H. and Liu, L. (2019) A CRISPR/LbCas12a-based method for highly efficient multiplex gene editing in Physcomitrella patens. *The Plant journal : for cell and molecular biology*, **100**, 863-872.

55. Sansbury, B.M., Wagner, A.M., Nitzan, E., Tarcic, G. and Kmiec, E.B. (2018) CRISPR-directed in vitro gene editing of plasmid DNA catalyzed by Cpf1 (Cas12a) nuclease and a mammalian cell-free extract. *The CRISPR journal*, **1**, 191-202.
